# Interferon-γ Regulates the Proliferation and Differentiation of Mesenchymal Stem Cells via Activation of Indoleamine 2,3 Dioxygenase (IDO)

**DOI:** 10.1371/journal.pone.0014698

**Published:** 2011-02-16

**Authors:** Juliana Croitoru-Lamoury, Francois M. J. Lamoury, Michael Caristo, Kazuo Suzuki, David Walker, Osamu Takikawa, Rosanne Taylor, Bruce J. Brew

**Affiliations:** 1 St. Vincent's Centre for Applied Medical Research, St. Vincent's Hospital, Sydney, Australia; 2 Department of Neurology, St. Vincent's Hospital, Sydney, Australia; 3 Department of Physiology, Monash University, Clayton, Australia; 4 Laboratory of Radiation Safety, National Institute for Longevity Sciences, National Center for Geriatrics and Gerontology, Morioka, Japan; 5 Faculty of Veterinary Science, University of Sydney, Sydney, Australia; Boston University School of Medicine, United States of America

## Abstract

The kynurenine pathway (KP) of tryptophan metabolism is linked to antimicrobial activity and modulation of immune responses but its role in stem cell biology is unknown. We show that human and mouse mesenchymal and neural stem cells (MSCs and NSCs) express the complete KP, including indoleamine 2,3 dioxygenase 1 (IDO) and IDO2, that it is highly regulated by type I (IFN-β) and II interferons (IFN-γ), and that its transcriptional modulation depends on the type of interferon, cell type and species. IFN-γ inhibited proliferation and altered human and mouse MSC neural, adipocytic and osteocytic differentiation *via* the activation of IDO. A functional KP present in MSCs, NSCs and perhaps other stem cell types offers novel therapeutic opportunities for optimisation of stem cell proliferation and differentiation.

## Introduction

In mammalian tissues and organs, including the brain, the kynurenine pathway (KP) is the central route that accounts for the degradation of the essential amino acid tryptophan (Trp) and ultimately generates the ubiquitous co-factor nicotinamide adenine dinucleotide (NAD+), which participates in basic cellular processes [1]. Named after a pivotal metabolite, kynurenine (KYN), the KP is a metabolic cascade of enzymatic steps, which yields several neuroactive compounds including quinolinic acid (QUIN), an N-methyl-D-aspartate (NMDA) receptor agonist that has neurotoxic effects [1]. The levels of these metabolites are determined by several KP enzymes, which in the brain are primarily contained in microglial cells and astrocytes (***Fig. S1***) [2]. The first and rate-limiting KP enzyme is indoleamine 2,3 dioxygenase (IDO), which has two isoforms IDO1 and IDO2 [3], [4] that can catabolize a similar range of substrates but with different efficiencies, and distinct responses to inhibitors [5], [6], [7]. Type II interferon IFN-γ is the key regulator of the KP [8], whereas other cytokines (TNF-α, IL-1, type I interferons IFN-α and IFN-β) or lipopolysaccharides are also able to induce IDO activation, although to a much lesser extent [9]. Additionally, a third KP enzyme, tryptophan 2,3 dioxygenase (TDO), catalyses the first step of the KP, and has been described as a homeostatic enzyme involved in the control of the basal levels of tryptophan in the serum. Unlike IDO, TDO does not respond to immunological signals and is primarily but not exclusively confined to the liver (for review [10]).

Involvement of the KP occurs in a variety of central nervous system disorders, infection and inflammatory conditions including multiple sclerosis (MS), AIDS-dementia complex and malaria [1], [11], [12]. More recently, the KP has been found to be an important determinant of immune responses and regulatory T-cell tolerance. This is thought to be related primarily to the activation of IDO and subsequent depletion of Trp in the microenvironment leading to impairment of protein synthesis and compromise of cell division [13] and/or the suppression of cell proliferation by a number of downstream KP metabolites such as QUIN [14], [15], [16]. Additionally, emerging evidence has shown that expression of IDO is required for the maturation of immune cells e.g. dendritic cells and regulatory T cells [17], [18]. Altogether these findings support the hypothesis that IDO activity in cells expressing the KP enzymatic machinery may play a critical role in modulating proliferation and differentiation in both a paracrine and autocrine manner.

Mesenchymal stem cells (MSCs) represent promising tools for the treatment of a wide range of neurological disorders and other degenerative diseases including MS, stroke, spinal cord injury and heart disease [19]. In addition to their ability to differentiate into osteoblasts, adipocytes, myoblasts, and controversially into neural cells, a number of recent studies have thrown new light on their unique immunomodulatory properties and possible therapeutic use [20]. Indeed, MSCs express the major KP enzyme IDO in response to IFN-γ [21], and thus can inhibit T cell proliferation and modulate the function of major cell populations involved in both the innate and adaptive immune systems, including antigen presenting cells, natural killer cells, T- and B-cells [19]. Furthermore, IFN-γ-induced activation of IDO in MSCs can create a Trp-depleted milieu that promotes immunosuppression and ameliorates experimental autoimmune encephalomyelitis (EAE), an animal model of MS [20], [22], [23]. Similar to MSCs, neural stem cells (NSCs) have also been shown to possess immunomodulatory functions as evidenced by studies showing that transplanted NSCs reduce brain inflammation in acute and chronic EAE [24], [25]. Of particular relevance to this is the observation that synthetic Trp metabolites are capable of suppressing proliferation of myelin-specific T cells and reversing paralysis in mice with EAE [26]. However, the mechanism by which MSCs and NSCs modulate the immune responses has not been completely resolved. More generally, the role of IDO and KP activity in stem cell biology and the exact nature of underlying mechanisms controlling stem cell proliferation and differentiation are completely unknown.

Here we describe a novel biological role for the KP and report that IFN-γ-induced IDO activation in MSCs leads to impaired proliferation and an alteration of their differentiation capacity. Our findings have immediate relevance to the optimization of MSCs in therapies for a wide range of diseases and raise the possibility that the selective manipulation of the KP in stem cells in general may represent a valuable therapeutic target.

## Results

### RNAs encoding all Kynurenine Pathway Enzymes are expressed in human and mouse MSCs under basal culture conditions

In the absence of any pro-inflammatory agents, quantitative real-time RT-PCR (qRT-PCR) revealed that human MSCs express low levels of RNAs encoding all KP enzymes, namely the major and rate limiting KP enzyme indoleamine 2,3-dioxygenase (IDO), tryptophan 2,3-dioxygenase (TDO), kynurenine formamidase (AFMID), tryptophan 5-monooxygenase 1 and 2 (TPH1 and TPH2), kynurenine aminotransferases I and II (CCBL1 and AADAT respectively), kynureninase (KYNU), kynurenine hydroxylase (KMO), 3-hydroxyanthranilate 3,4 dioxygenase (HAAO) and aminocarboxymuconate-semialdehyde decarboxylase (ACDMS) and quinolinate phosphoribosyltransferase (QPRT) (***Table 1***). The levels of expression (i.e. the ratio gene/β-actin) of RNAs encoding the KP enzymes in unstimulated human MSCs were comparable to those observed in human total brain tissue or purified astrocytes but lower than in unstimulated macrophages (***Table 1***). In particular, the gene/β-actin ratios for KYNU, KMO, CCBL1 and ACDMS were significantly lower in human MSCs than in macrophages. Similar results were obtained with mouse MSCs, when the gene/β-actin ratios were compared to the expression of KP enzymes in mouse tissues including the brain, testis, kidney, liver, thymus, spleen and PBMCs (***Tables 1*** ***, S2***). Additionally, we provide the first evidence that mouse NSCs express transcripts encoding all KP enzymes under basal conditions of culture (***Tables 1*** ***, S2***).

**Table 1:** Quantitative real–time RT-PCR analysis of total RNA encoding the kynurenine pathway enzymes in mouse and human MSCs cultured in the absence or presence of IFN-γ (100 IU/ml) or IFN-β (2,000 IU/ml) for 72 hours.

		hMSCsControl	hMSCsIFN-γ 100 IU/ml	hMSCsIFN-β 2000 IU/ml	MΦControl	MΦIFN-γ 100 IU/ml	mMSCs Control	mMSCsIFN-γ 100 IU/ml	mMSCsIFN-β 2000 IU/ml	mPBMCsControl	mPBMCsIFN-γ 100 IU/ml	mNSCsControl	mNSCsIFN-γ 100 IU/ml
**Full IDO1**	Mean	0.0290	11,865.0000	57.0000	0.0000	359.5000	0.0001	5.8600	0.0000	0.0000	0.0251	0.1584	0.0660
	SD	0.0000	403.0509	0.4243	0.0000	7.7782	0.0002	0.3818	0.0000	0.0000	0.0028	0.0102	0.0112
**Partial IDO1**	Mean	0.0012	1,442.5000	2.1250	0.0000	56.5000	0.0000	4.7200	0.0634	0.0000	0.0266		
	SD	0.0000	6.3640	0.0071	0.0000	4.6669	0.0000	0.1273	0.0266	0.0000	0.0019		
**Full IDO2**	Mean	0.0047	0.3580	0.0395	15.3500	0.0323	0.0000	0.0000	0.0000	0.0000	0.0000	0.6134	0.5784
	SD	0.0020	0.0014	0.0011	0.3536	0.0456	0.0000	0.0000	0.0000	0.0000	0.0000	0.0370	0.0617
**Partial IDO2**	Mean	0.0073	0.8985	0.0882	44.1000	0.9595	0.2050	8.2150	0.2590	0.1760	0.1865		
	SD	0.0103	0.0672	0.0035	6.9296	0.1421	0.0057	0.1768	0.0552	0.0085	0.0205		
**KYNU**	Mean	0.3750	9.5450	4.1500	399.0000	287.0000	0.0001	0.0000	0.0000	0.6765	3.3150	0.0113	0.0000
	SD	0.0481	0.4455	0.2546	53.7401	8.4853	0.0000	0.0000	0.0000	0.0134	1.0677	0.0012	0.0000
**ACDMS**	Mean	0.4490	0.1265	0.5365	11.3300	0.3270	0.0024	0.0000	0.0037				
	SD	0.0651	0.0007	0.1619	3.6345	0.2263	0.0008	0.0000	0.0000				
**TPH1**	Mean	0.4755	0.4775	1.2200	6.4550	0.1290	0.1865	0.1285	0.2225				
	SD	0.0389	0.0049	0.0566	2.6375	0.0170	0.0134	0.0049	0.0304				
**TPH2**	Mean	0.0089	0.0079	0.0523	0.0031	0.6117	0.0746	0.1290	0.0449			0.1032	4.6638
	SD	0.0038	0.0029	0.0088	0.0002	0.8179	0.0023	0.0000	0.0049			0.0110	2.1664
**KMO**	Mean	0.0187	0.1081	1.4000	10.5300	178.5000	0.0355	0.0387	0.0453	0.1780	0.1616	0.0190	0.0000
	SD	0.0258	0.0268	0.1131	1.5132	3.5355	0.0087	0.0012	0.0124	0.0636	0.0981	0.0020	0.0000
**HAAO**	Mean	0.2610	0.8015	0.8870	2.1500	41.1000	0.4500	0.3005	0.3300	0.5055	0.4910	0.0000	0.0573
	SD	0.0665	0.0346	0.1146	0.0990	0.4243	0.0014	0.0092	0.0339	0.0276	0.0354	0.0000	0.0266
**CCBL1**	Mean	3.0750	1.6500	4.0700	12.1500	1.7400	9.6200	5.4800	7.2950	4.6600	5.2850	10.0503	39.1429
	SD	0.4596	0.1131	0.3818	0.4950	0.1273	0.1273	0.0141	0.2899	0.3394	0.1909	0.7898	18.1827
**AFMID**	Mean	0.4990	0.6870	0.8205	0.0016	0.2430	7.3350	7.6850	5.8800	0.9800	0.8295	0.2441	0.7937
	SD	0.0339	0.0042	0.1520	0.0023	0.0410	0.0778	0.3606	0.1414	0.0042	0.1421	0.0260	0.3687
**QPRT**	Mean	0.2165	0.0779	0.7325	0.1233	0.9735	7.4800	7.8900	6.4350	0.8135	0.8105	0.5222	38.2592
	SD	0.0601	0.0131	0.0672	0.1056	0.0233	0.0424	0.4950	0.5020	0.0714	0.1365	0.0557	17.7723
**AADAT**	Mean	20.0000	18.5000	35.1000	23.3000	0.6275	0.2090	0.1710	0.2045	0.0147	0.0233	6.1594	60.8252
	SD	0.2828	0.1414	0.7071	0.9899	0.0884	0.0495	0.0057	0.0714	0.0013	0.0013	0.6572	28.2547
**TDO2**	Mean	0.1800	0.1830	1.0920	3.1900	2.3250	0.0109	0.0142	0.0137	0.0000	0.0031	0.0205	0.0429
	SD	0.0424	0.0198	0.1386	0.1556	0.4455	0.0081	0.0075	0.0075	0.0000	0.0001	0.0012	0.0046

The gene/β-actin ratios were multiplied by 10,000 for clarity purposes. Data are mean ± standard deviation (SD). Abbreviations: IFN-γ, interferon-γ; IFN-β, interferon-β; MSCs, mesenchymal stem cells; PBMCs, peripheral blood mononuclear cells; IDO1 and IDO2, indoleamine 2,3-dioxygenase 1 and 2; KYNU, kynureninase; ACDMS, aminocarboxymuconate-semialdehyde decarboxylase; TPH1 and TPH2, tryptophan 5-monooxygenase 1 and 2; KMO, kynurenine hydroxylase; HAAO, 3-hydroxyanthranilate 3,4 dioxygenase; CCBL1 and AADAT, kynurenine aminotransferases I and II respectively; AFMID, kynurenine formamidase; QPRT, quinolinate phosphoribosyltransferase; TDO2, tryptophan 2,3-dioxygenase.

Indoleamine 2,3-dioxygenase-2 (IDO2), also referred to as indoleamine 2,3-dioxygenase-like protein (INDOL1), has been recently described [4], [5]. IDO2 is structurally similar to IDO1; it can use a similar range of substrates but differs in the selectivity for some IDO inhibitors [4], [5] - norharmane is IDO1- and IDO2-specific, while L- and D-isomers of 1-methyl tryptophan inhibitors are specific of IDO1 and IDO2 respectively. The genes encoding IDO1 and IDO2 utilize multiple promoters to express transcripts of different lengths and generate both active and truncated proteins [4], [5]; their expression in stem cells remains to be characterized.

**Figure 1:**
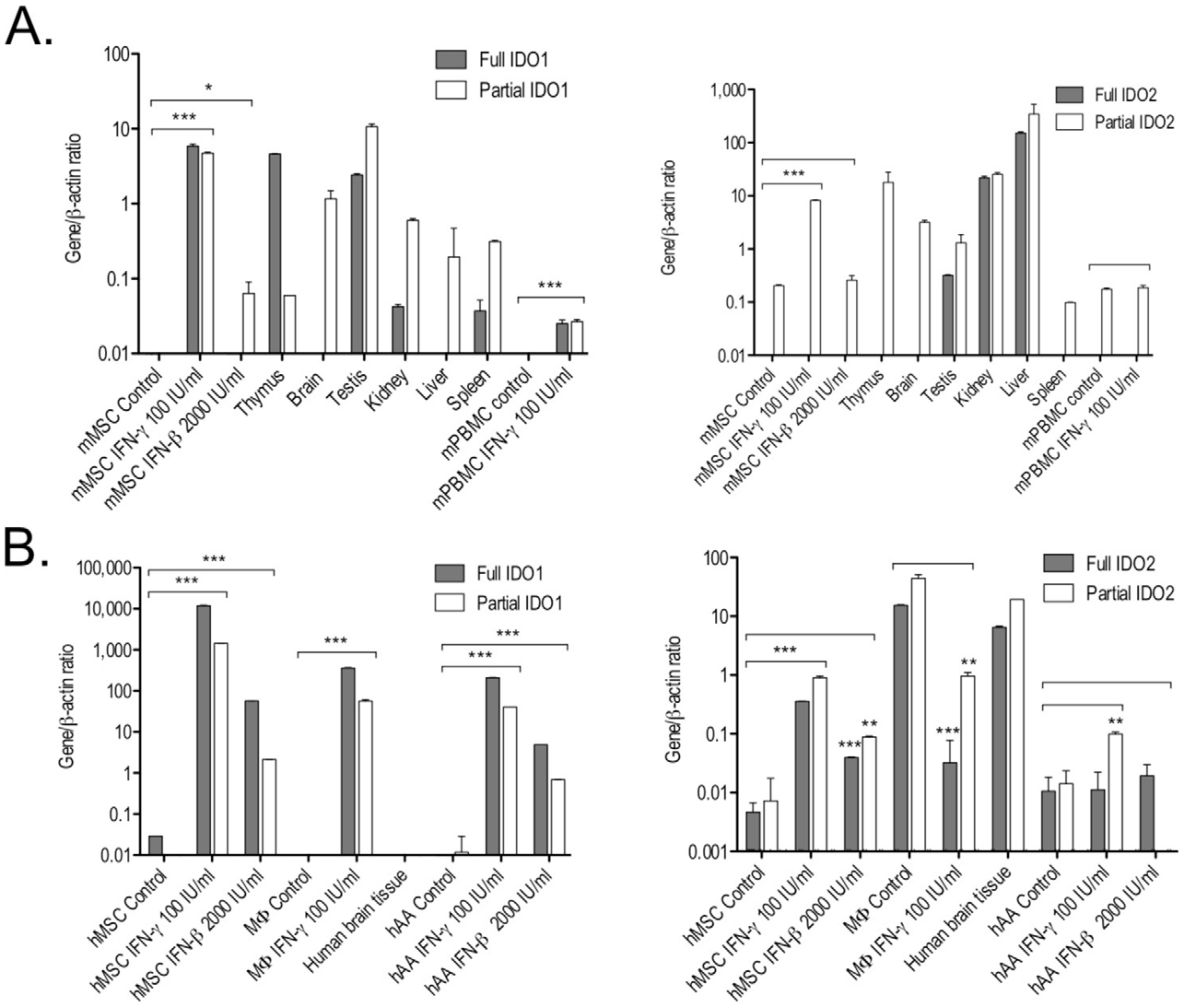
Expression of full and partial IDO1 and IDO2 by human and mouse MSCs and various tissues as measured by quantitative reverse transcription polymerase chain reaction (qRT-PCR). Human and mouse MSCs at passages 6 and 21 respectively were cultured in the absence or presence of IFN-γ (100 IU/ml) or IFN-β (2,000 IU/ml) for 72 hours. **A:** Mouse cells and tissues. **B:** Human cells and tissues. The gene/β-actin ratios were multiplied by 10,000 for clarity purposes. Data are mean ± standard deviation (SD). *p<0.05, **p<0.01, ***p<0.001 when compared with control (without cytokine treatment). Differences between two groups were analyzed by the two-tailed Student's *t*-test and of more than two groups by one-way ANOVA with *post-hoc* Dunnett's Multiple Comparison test. **Abbreviations:** IFN-γ, interferon-γ; IFN-β, interferon-β; MSCs, mesenchymal stem cells; PBMCs, peripheral blood mononuclear cells; hAA, human adult astrocytes; IDO1 and IDO2, indoleamine 2,3-dioxygenase 1 and 2; MΦ, macrophages.

Therefore, we optimized a qRT-PCR method to discriminate active and truncated IDO transcripts and examined their expression in both mouse and human MSCs. We demonstrate that unstimulated mouse MSCs express detectable levels of transcripts encoding full IDO1 and partial IDO2 genes, but no full IDO2 or partial IDO1 RNAs (***Table 1***, ***Fig. 1A***, ***Fig. S2***).

Significant positive fold changes in RNA expression of partial IDO2 (40 times), full IDO1 (44,000 times) and partial IDO1 were detected after stimulation of mouse MSCs with 100 IU/ml IFN-γ over 72 hours (***Table 1***, ***Fig. 1A***). However, full IDO2 transcripts were not detectable in mouse MSCs at significant levels even after IFN-γ treatment, suggesting that, unlike IDO1, IDO2 gene activity is not dependent on IFN-γ and/or might not be translated into a full, functional protein in these cells.

In line with observations from Ball *et al*. [4], [5], the highest levels of IDO2 mRNA were observed in the mouse liver, followed by the kidney, testis and brain tissues. There was no significant expression of full IDO1 transcripts, and only partial IDO1 and IDO2 RNAs were detected in the healthy mouse brain (***Table 1***, ***Fig. 1A***, ***Table S2***).

In contrast to mouse MSCs, unstimulated human MSCs can express both full and partial mRNAs encoding IDO1 and IDO2 genes (***Table 1***, ***Fig. 1B***, ***Table S2***). Moreover, the levels of expression of both genes were significantly up-regulated after exposure of human MSCs to IFN-γ over 72 hours. Indeed, the analysis by qRT-PCR of IDO1 and IDO2 genes after exposure of human MSCs to 100 IU/ml IFN-γ revealed an increase of 409,000 and 76 times (for full transcripts) and 1,227,659 and 123 times (for partial transcripts) respectively (***Table 1***, ***Fig. 1B***).

Interestingly, unstimulated human macrophages do not express detectable levels of full or partial IDO1 transcripts but only full and partial IDO2 and TDO RNAs (***Table 1***, ***Fig. 1B***). However, after treatment of macrophages with 100 IU/ml IFN-γ over 72 hours, expression of full and partial IDO1 transcripts was observed and interestingly, a significant decrease of full and partial IDO2 RNAs.

### IFN-γ is a key regulator of the expression of the kynurenine pathway enzymes in human and mouse MSCs

**Figure 2:**
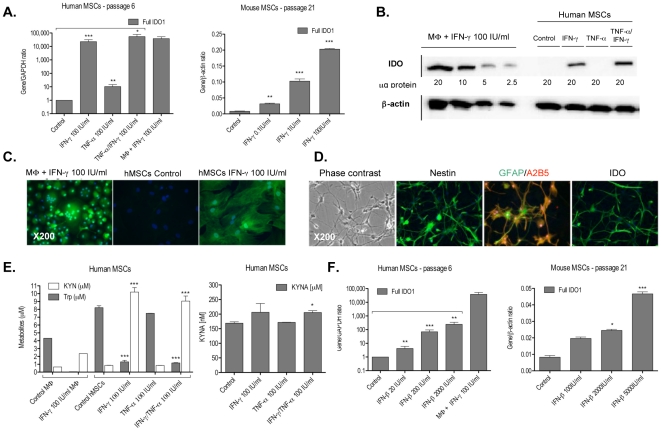
IFN-γ and IFN-β are responsible for IDO induction at the gene and protein level in human and mouse stem cells. Human and mouse MSCs were cultured in the absence or presence of TNF-α (100 IU/ml) and/or IFN-γ (0.1, 1 or 100 IU/ml) or IFN-β (100, 2,000 or 5,000 IU/ml) for 72 hours. Data are mean ± standard error (SEM). **Abbreviations:** TNF-α, tumour necrosis factor-α; IFN-γ, interferon-γ; IFN-β, interferon-β; GAPDH: glyceraldehyde-3-phosphate dehydrogenase; MSCs, mesenchymal stem cells; IDO1 and IDO2, indoleamine 2,3-dioxygenase 1 and 2; MΦ, macrophages; Trp, tryptophan; KYN, kynurenine; KYNA; kynurenic acid; GFAP, glial fibrillary acidic protein. **A.** Gene expression of full IDO1 in human and mouse MSC cultures at passages 6 and 21 respectively as measured by qRT-PCR. **B.** Western blot analysis of IDO1 protein in human MSCs cultured with cytokines for 72 hours. β-actin was used as loading control. **C.** Immunocytochemistry analysis of IDO protein expression in human MSCs. Macrophages were used as positive control. Magnification X200. **D.** Fluorescent immunocytochemical labelling for IDO and neural proteins in mouse NSCs after 72 hours of culture in the presence of 100 IU/ml IFN-γ and neural induction media (magnification X200). **E.** Tryptophan degradation, kynurenine and kynurenic acid production by human MSCs and macrophages measured by HPLC 72 hours after stimulation with cytokines. **F.** Full IDO1 mRNA expression of 72 hours stimulation of human and mouse MSCs with IFN-β (passages 6 and 21 respectively) as measured by qRT-PCR. *p<0.05, **p<0.01, ***p<0.001 when compared with control (without cytokine treatment). Differences between two groups were analyzed by the two-tailed Student's *t*-test and of more than two groups by one-way ANOVA with *post-hoc* Dunnett's and Tukey's Multiple Comparison test.

To further examine the effects of pro-inflammatory cytokines on the regulation of KP activity in MSCs, cultures of mouse and human MSCs and mouse NSCs were exposed to TNF-α and/or IFN-γ for up to 72 hours. Total RNA was extracted and qRT-PCR analysis was performed at 72 hours post-stimulation (***Table 1***, ***Fig. 2***, ***Table S2***).

We demonstrate that in human and mouse MSC and mouse NSC cultures, IFN-γ was responsible for modulation of IDO at both the gene and protein levels and its effect was dose-dependent after 3 days in culture as revealed by qRT-PCR, western blot analysis and immunocytochemistry (***Table 1***, ***Fig. 2A–D***, ***Table S2***).

In addition, IFN-γ treatment of human MSCs induced marked changes of other KP enzymes at the RNA level (***Table 1***). In the presence of IFN-γ, IDO1 (409,000 times), and to a lesser extent IDO2, KYNU, KMO and HAAO gene expression were significantly up-regulated, while the expression of distal KP enzymes AADAT, CCBL1, ACDMS and QPRTase were down-regulated by IFN-γ (***Table 1***). No significant effect of IFN-γ was noted for the expression of TPH1, AFMID and TDO genes. Thus, these results suggest that activation of the KP by IFN-γ in human MSCs favours the production of anthranilic acid and QUIN, at the expense of kynurenic acid synthesis. This was confirmed by HLPC, which demonstrated that upon stimulation with 100 IU/ml IFN-γ human MSCs were able to catabolize tryptophan, and release kynurenine but not kynurenic acid (***Fig. 2E***).

In comparison to human MSCs, IFN-γ-stimulated macrophages show higher levels of induction of AFMID, KMO, TPH2, HAAO and QPRT gene expression, and surprisingly, a lower level of activation of IDO1, together with a down regulation of IDO2, KYNU, TPH1, AADAT, CCBL1 and ACDMS (***Table 1***). Notably, discrepancies were observed for the regulation of IDO2 and QPRT genes in MSCs and macrophages, demonstrating that IFN-γ effects on KP expression are cell-specific.

Surprisingly, in mouse MSCs IFN-γ did not cause significant changes in the expression of KP enzymes at the RNA level, with the exception of IDO1, IDO2 (as described above) and KYNU, which indicates that the regulation of the KP by IFN-γ is also species-specific.

### IFN-β modulates the gene expression of Kynurenine pathway enzymes in MSCs

Next, we investigated the effects of the type I interferon IFN-β on the activation of the KP in human and mouse MSCs. After stimulation of human MSC cultures with IFN-β for 72 hours, full IDO1, partial IDO1, partial IDO2, KMO and KYNU gene expression was significantly up-regulated (1,965, 1,808, 12, 74 and 11 times respectively) while the increase in the expression of other transcripts i.e. full IDO2, TDO2, AFMID, CCBL1, AADAT, HAAO, ACDMS, QPRT was more modest (***Table 1***, ***Fig. 1***, ***Table S2***). Interestingly, while IFN-β treatment activated the expression of full and partial IDO1, KYNU, KMO, TPH1, TPH2 and TDO2 genes in human adult astrocytes, ACDMS, HAAO, CCBL1, AFMID and AADT mRNA was down-regulated. This indicates that like IFN-γ, IFN-β's effects on KP expression are cell-specific.

Surprisingly, the levels of expression of most KP enzymes in mouse MSCs were down-regulated following treatment with IFN-β, in particular a decrease of the full IDO1 mRNA (***Table 1***, ***Table S2***). Hence, these results reveal a differential modulation of IFN-β-induced expression of KP enzymes between species.

### IFN-γ inhibits the proliferation of mouse and human mesenchymal stem cells through activation of the KP

**Figure 3:**
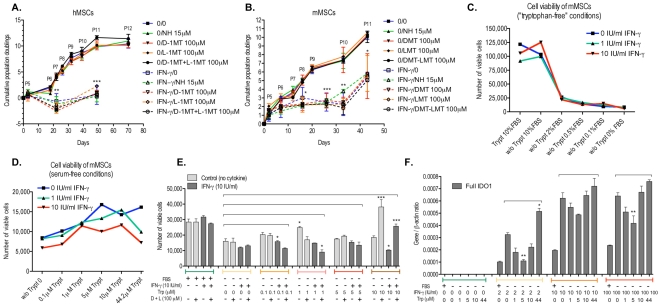
IFN-γ inhibits the proliferation of mouse and human MSCs. **A** and **B**. Cell growth of human and mouse MSCs showing cumulative population doublings as a function of time in culture. Between P4 and P12, cells were cultured in the continuous presence of IFN-γ (100 IU/ml) and/or IDO inhibitors norharmane, D-1-methyl-tryptophan and L-1-methyl-tryptophan for 80 and 50 days respectively. At every passage, population doubling was calculated by the formula logN/log2 as described by Stenderup [63] where N is the ratio between the number of viable cells reaching confluence and the number of cells initially plated. Medium was changed every three days using α-MEM containing 2 mmol/L L-glutamine, 100 units/mL penicillin, 100 mg/mL streptomycin, 20% or 10% non-inactivated FBS, for human and mouse cells respectively, specially tested for the ability to sustain the growth of MSCs. **C.** Number of viable cells as measured by Alamar blue in mouse MSC cultures (passage 29) grown in DMEM F12 medium without tryptophan and treated for 5 days with increasing concentrations of FBS (0, 0.1, 0.5, 2 or 10%) and IFN-γ (0, 1 or 10 IU/ml) **D.** Number of viable cells as measured by Alamar blue in mouse MSC cultures (passage 29) grown in DMEM F12 medium without serum and treated for 5 days with increasing concentrations of tryptophan (0, 0.1, 1, 5, 10 or 44.2 µM) and IFN-γ (0, 1 or 10 IU/ml) **E.** Number of viable mouse MSCs (passage 14) cultured in serum free DMEM F12 medium in the presence of 10 IU/ml IFN-γ, increasing concentrations of tryptophan (0, 0.1, 1, 5, and 10 µM) and/or IDO inhibitors D-1-methyl-tryptophan and L-1-methyl-tryptophan (100 µM) for 5 days. Mouse MSCs were cultured with 10% FBS as positive controls. **F.** Expression of full IDO1 mRNA in mouse MSCs (passage 20) as measured by qRT-PCR. Cells were grown in the presence of increasing concentrations of tryptophan (0, 1, 5, 10 and 44 µM) and/or IFN-γ (0, 2, 10 and 100 IU/ml) for 24 hours. Mouse MSCs were cultured with 10% FBS as positive controls. Data are mean ± standard error (SEM). *p<0.05, **p<0.01, ***p<0.001 when compared with control (without cytokine or KP inhibitors treatment). Differences between two groups were analyzed by the two-tailed Student's *t*-test and of more than two groups by one-way ANOVA with *post-hoc* Dunnett's Multiple Comparison test. **Abbreviations:** IFN-γ, interferon-γ; MSCs, mesenchymal stem cells; IDO1, indoleamine 2,3-dioxygenase 1; Trp, tryptophan; NH, norharmane; D-1MT, D-1-methyl-tryptophan; L-1MT, L-1-methyl-tryptophan; FBS, foetal bovine serum; F12, DMEMF12.

To determine whether IFN-γ may affect the proliferation of human and mouse MSCs through the activation of the KP and subsequent depletion of tryptophan from the extracellular milieu, cells were cultured in α-MEM containing 20% and 10% FBS respectively, in the presence of 100 IU/ml of IFN-γ; cell growth was then assessed in long-term cultures. ***Figure 3A*** shows a significant decrease of the proliferative response of IFN-γ-treated human MSCs from Day 22 (94% inhibition) when compared to the controls, which was not reversed by the addition of IDO inhibitors norharmane (NH), L-1-methyl-tryptophan (L-1MT) and/or D-1-methyl-tryptophan (D-1MT) in the culture medium (***Fig. 3A***). Alterations in the levels of cell growth were comparable in mouse MSCs, although equivalent levels of inhibition were attained after 33 days of culture (***Fig. 3B***). In our experimental conditions, the results indicate that in long-term cultures: a) the effects mediated by IFN-γ on the proliferation might not be exclusively associated with IDO activity in MSCs and/or b) the tryptophan originating from the FBS available to the cells was in excess when compared to the amount catabolized by MSCs.

Therefore, in order to distinguish the effects of IFN-γ and tryptophan on the cell growth of mouse MSCs, we performed experiments using custom made tryptophan-free media and increasing concentrations of serum (0 to 10% FBS) or tryptophan (0 to 44.2 µM). After 24 hours of culture, recombinant mouse IFN-γ was added at a concentration of 0, 1 and 10 IU/ml. As shown in ***Figure 3C*** *and* ***Figure S3***, mouse MSCs are still viable after 5 days in the culture medium without tryptophan and serum but cell growth is compromised. Cells grown in 2% serum but without additional tryptophan were inhibited to 46% and 42%, respectively, of the control or the regular DMEMF12 10% FBS culture conditions. Addition of IFN-γ at concentrations of 1 and 10 IU/ml resulted in no significant alteration of the cell growth of mouse MSCs grown in the medium without tryptophan (***Fig. 3C***).

Conversely, for cultures of mouse MSCs grown in the absence of serum, 10 IU/ml of IFN-γ added simultaneously with 5 µM of tryptophan was required to obtain 25% inhibition of cell growth (***Fig. 3D***). The maximum blockage of the anti-proliferative activity of IFN-γ was obtained in mouse MSCs when 44.2 µM of tryptophan (concentration in the regular medium) was added to the culture. The cell growth was proportionally compromised with the increase of tryptophan concentration in the culture medium, supporting IFN-γ's effects on the proliferation of mouse MSCs being directly associated with KP-mediated tryptophan depletion. Indeed, blocking the IDO enzymatic activity using the specific inhibitors D-1MT and L-1MT re-established the cell growth of mouse MSCs cultured in the presence of high concentrations of tryptophan (10 µM) (***Fig. 3E***), further supporting the hypothesis that IFN-γ anti-proliferative effects are specifically linked with IDO activity in these stem cells.

We also demonstrate that the concentration of tryptophan is responsible for the transcriptional regulation of IDO1 (***Fig. 3F***). For a given concentration of IFN-γ, the IDO mRNA levels were down-regulated at low concentrations of tryptophan (0 to 5 µM), while high concentrations of tryptophan (10 and 44 µM) significantly increase the IDO1 gene transcription levels (***Fig 3F***). Interestingly, MSCs cultured in the presence of FBS and IFN-γ displayed lower IDO1 gene expression levels than cells grown in serum-free medium (***Fig. 3F***).

In an effort to elucidate whether the suppressive effects of IFN-γ on the proliferation of MSCs were solely due to IDO activation and the subsequent depletion of extracellular tryptophan, we further investigated the ability of MSC i) to release toxic KP metabolites i.e. QUIN that might hinder cell proliferation and ii) to express WRS, STAT1 and PI3K, three genes involved in the control of intracellular tryptophan levels [10], [27], [28]. Surprisingly, significant QUIN production by MSCs was not detectable in our experimental conditions, even after the treatment with IFN-γ (data not shown). However, we show that unstimulated human and mouse MSCs express transcripts encoding WRS, STAT1 and PI3K, and also that IFN-γ and to a lesser extent IFN-β are potent inducers of WRS and STAT1 expression (***Fig. S4A,B***, ***Table S2***). Similar to IDO1, for a given concentration of IFN-γ, WRS and STAT transcriptional activity in MSCs was modulated by the concentration of tryptophan (***Fig. S4C***). At the physiological level, this indicates that IFN-γ's effect on cell proliferation in MSCs is correlated to both the amount of tryptophan available and the level of IDO and WRS activity.

### IFN-γ and IFN-β modulate the differentiation potential of mouse and human mesenchymal stem cells through the activation of the KP

Given our findings that IFN-γ inhibited the proliferation of MSCs through activation of IDO, we hypothesised that IFN-γ might also affect the differentiation potential of these stem cells *via* similar mechanisms i.e. the activation of the KP. *In vitro* differentiation of both human and mouse MSCs into neural cells, adipocytes and osteocytes was performed in the presence of IFN-γ and IDO1 and IDO2 inhibitors.

#### Differentiation of MSCs into osteoblastic and adipogenic lineages

**Figure 4:**
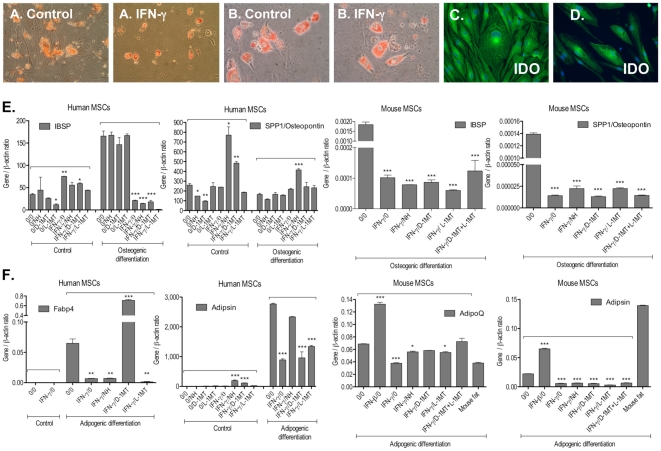
IFN-γ modulates the osteogenic and adipogenic differentiation potential of human and MSCs. Human and mouse MSCs were maintained in osteogenic (A, C) and adipogenic (B, D) differentiation media as described in *Material and Methods*. **A.** Representative photographs of differentiated osteocytes derived from control and IFN-γ-stimulated hMSCs. The osteocytic differentiation is demonstrated by Alizarin Red staining (magnification X40). **B.** Visualisation of lipid vacuoles in differentiated adipocytes derived from control and IFN-γ-stimulated mMSCs as revealed by Oil Red O staining (magnification X40). **C.** and **D.** Fluorescent immunocytochemical labelling for the IDO protein in differentiating hMSCs after 14 days of culture in the presence of 100 IU/ml IFN-γ and osteogenic (C) and adipogenic (D) induction media respectively (magnification X200). **E.** and **F.** Gene expression of (E) osteoblastic (osteopontin, IBSP - integrin-binding sialoprotein II) and (F) adipocytic (adipsin, adipoQ, Fabp4) markers in human and mouse MSC cultures after 3 and 7 days of differentiation procedures respectively as measured by qRT-PCR. Human MSCs (passage 12) and mouse MSCs (passage 13) were cultured in the induction media together with 100 IU/ml IFN-γ and/or IDO inhibitors norharmane (15 µM), D-1-methyl-tryptophan (100 µM) and L-1-methyl-tryptophan (100 µM). Data are mean ± standard error (SEM). *p<0.05, **p<0.01, ***p<0.001 when compared with control (no treatment). Differences between two groups were analyzed by the two-tailed Student's *t*-test and of more than two groups by one-way ANOVA with *post-hoc* Dunnett's and Tukey's Multiple Comparison test. **Abbreviations:** IFN-γ, interferon-γ; MSCs, mesenchymal stem cells; IDO, indoleamine 2,3-dioxygenase; NH, norharmane; D-1MT, D-1-methyl-tryptophan; L-1MT, L-1-methyl-tryptophan; IBSP, integrin-binding sialoprotein II; SPP1, secreted phosphoprotein 1; Fabp4, fatty acid binding protein 4.

To confirm multipotentiality of MSCs, we assessed their ability to differentiate into cells of osteogenic and adipogenic lineages. Prior to differentiation experiments, fluorescent activated cell sorting (FACS) analysis confirmed that the expanded, plastic adherent cells were positive for the surface markers CD73 and CD90, but negative for CD11b, CD19, CD34, CD45 and HLA-DR (data not shown). Next, MSCs were placed in induction media specific for the generation of adipocytes and osteocytes according to our published protocol [29]. Lipid vacuoles in differentiated adipocytes were visualised with Oil Red O (***Fig. 4B***) while the osteogenic differentiation of MSCs was demonstrated by Alizarin Red staining (***Fig. 4A***).

As previously published by our laboratory, qRT-PCR showed that mouse and human MSCs maintained under basal conditions constitutively express osteoblastic and adipocytic markers - osteopontin (SPP1), integrin-binding sialoprotein II (IBSP) and adipsin, adipoQ, Pparg respectively (***Fig. 4E,F***) - and the expression of each greatly increased after induction [29]. We also demonstrate here that treatment with 100 IU/ml IFN-γ resulted in the inhibition of SPP1 and IBSP gene expression and the subsequent osteocytic differentiation of both mouse and human MSCs (***Fig. 4E***). Additionally, blocking the IFN-γ-induced IDO activity with the inhibitor norharmane (15 µM) significantly increased SPP1 gene expression, supporting the role of IDO in the control of the osteocytic differentiation potential of MSCs (***Fig. 4E***).

Similarly, IFN-γ inhibited the expression of the adipocytic markers adipsin, adipoQ and Fabp4 at the transcriptional level in mouse and human MSCs during the differentiation experiments (***Fig. 4F***). In agreement with our experimental paradigm that IDO activity in MSCs influences their adipocytic differentiation potential, inhibition of IDO by norharmane in MSCs led to an improved gene expression for adipsin and adipoQ, whereas DMT was effective on Fabp4 transcriptional activity. Notably, in contrast to IFN-γ, type I interferon IFN-β significantly *increased* the expression of adipsin and adipoQ transcripts in differentiated mouse MSCs.

#### Differentiation of MSCs into neural cells

**Figure 5:**
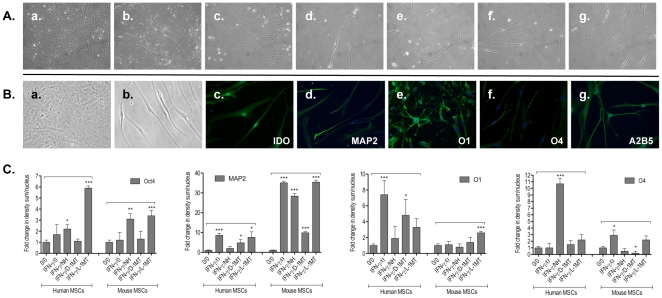
IFN-γ modulates the neural differentiation potential of human MSCs. **A.** Representative phase contrast photographs of human MSCs cultured for 14 days using the nestin induction medium then 7 days in neural differentiation medium as described in *Material and Methods* (Magnification X200). Cells were cultured in the neural differentiation media in the presence of 100 IU/ml IFN-γ and/or IDO inhibitors norharmane (15 µM), D-1-methyl-tryptophan (100 µM) and L-1-methyl-tryptophan (100 µM). **a:** Control, α-MEM, 20% FBS; **b:** 14 days with nestin induction medium; **c-g:** 7 days with neural differentiation medium; **c:** Control neural differentiation, no IFN-γ or IDO inhibitors; **d:** IFN-γ, no IDO inhibitors; **e:** IFN-γ, norharmane; **f:** IFN-γ, 1Methyl-D-Tryptophan; g: IFN-γ, 1Methyl-L-Tryptophan. **B.** Fluorescent immunocytochemical labelling for IDO and neural proteins in differentiating human MSCs after 7 days of culture in the presence of 100 IU/ml IFN-γ and neural differentiation medium (magnification X200). **a:** Undifferentiated human MSCs control, α-MEM, 20% FBS; **b:** Control neural differentiation; **c:** IDO, indoleamine 2,3-dioxygenase; **d:** MAP2, microtubule-associated protein 2; **e:** O1; **f:** O4; **g:** A2B5. Magnification X200. **C:** Quantification of immunostaining for precursor and neural markers in differentiating human MSCs after 7 days of culture in the presence of 100 IU/ml IFN-γ and neural differentiation medium. Images were taken under identical exposure conditions. Fold changes in the density of immunostaining normalised to the number of nuclei are represented as means ± SEM of at least three independent experiments. *p<0.05, **p<0.01, ***p<0.001 when compared with control (no treatment). Differences between two groups were analyzed by the two-tailed Student's *t*-test and of more than two groups by one-way ANOVA with *post-hoc* Dunnett's Multiple Comparison test.

Next, we investigated whether the neural differentiation potential of human and mouse MSCs could be affected by the type I and II interferons IFN-γ and IFN-β respectively, due to the activation of the KP. Thus, to induce the neural differentiation of MSCs, we optimised one media formulation as described in *Material and Methods* (***Fig. 5A,B***). Immunocytochemical analyses with antibodies specific for neural markers including A2B5, O1, O4, Olig1, glial fibrillary acidic protein (GFAP), microtubule-associated protein 2 (MAP2) and neuron specific enolase (NSE) indicated robust expression of several of these markers in MSCs after neural induction procedures (neuro-MSCs) (***Fig. 5B***, ***Fig. S5***).

In human neuro-MSCs at three different passages, cultured in the presence of interferons and/or IDO inhibitors, both IFN-γ and IFN-β caused significant changes at the transcriptional level for genes encoding a wide range of neural markers (*Table S3*). Addition of IFN-γ increased the levels of MAP2, CNP1, Hes1, and GRM1 mRNA following neural induction of MSCs and repressed the transcription of Id2, GalC, NG2, MOG, NPDC1 and SCLIA1 genes, whereas nestin, GFAP, NFkB and SCLAI3 expression remained unaffected. In most cases, these effects were counterbalanced by the addition of IDO inhibitors norharmane, DMT and/or LMT to the neural induction media, with the exception of nestin, Id2, CNP1, GFAP, SCLIA1 and SCLIA3 genes (***Table S3***), confirming that IDO activity in neuro-MSCs modulates their neural differentiation potential.

Interestingly, IFN-β significantly upregulated MAP2, CNP1 and SCLIA3 RNA, but had no effect on nestin, Hes and GFAP gene regulation in neuro-MSCs. In addition, the transcription levels of Id2, GalC, NG2, MOG, NPDC1, NFkB, GRM1 and SCLIA1 were down-regulated by addition of IFN-β to the neural induction media (***Table S3***).

To corroborate the involvement of IDO activation in this IFN-γ-mediated modulation of MSC neural differentiation potential of MSCs, the IDO inhibitors norharmane, D-1MT and L-1MT were used in conjunction with IFN-γ in the neural differentiation experiments and quantification of neural proteins expressed by neuro-MSCs was performed by immunocytochemistry (***Fig. 5C***, ***Table S4***). As expected, IFN-γ greatly enhanced IDO, MAP2, O1, O4, Oct4, A2B5 protein levels in differentiated mouse and human MSCs. Norharmane was the most effective of the IDO inhibitors, drastically attenuating the expression of IDO, O1, O4, MAP2 proteins by both human and mouse neuro-MSCs, while D-1MT had a less profound inhibitory effect. These data confirm that the IFN-γ-induced activation of IDO in both human and mouse MSCs specifically influences their neural differentiation potential. Interestingly, the addition of norharmane or LMT simultaneously with IFN-γ to the neural induction media resulted in a significant *increase* of the proportion of neuro-MSCs expressing the markers Oct 4 and A2B5, suggesting that these inhibitors might exert other cellular effects, in addition to the suppression of IDO activity.

## Discussion

In this investigation we have established that the KP is present and active in MSCs and NSCs, and demonstrated that IDO enzymatic activity is of critical importance in the control of proliferation and differentiation of MSCs. We showed that mouse and human MSCs express the complete, functional KP enzymatic machinery, including IDO1 and its recently identified paralogue IDO2 [4], [5], and that the expression of the KP enzymes is highly regulated by both type I and II interferons i.e. IFN-β and IFN-γ respectively. In addition to the recent findings that IDO1, IDO2 and TDO2 mRNA are expressed in human MSCs [30], we accomplished a comprehensive examination of the transcriptional regulation of full and truncated IDO1 and IDO2 transcripts in both mouse and human MSCs. Species-dependent differences in the expression and regulation of IDO paralogues were noted (e.g. significant levels of full IDO2 transcripts were not detectable in mouse MSCs even after IFN-γ treatment), which indicate that IDO activity may be dissimilar in human cells compared to rodent cells [6], [10].

We also report a differential transcriptional modulation of KP components depending on the type of interferon, cell types and species. Paradigmatic in this regard is the case of QPRT in MSCs and macrophages, where IFN-γ exerts opposite effects at the gene level. These results probably explain the absence of significant QUIN production by MSCs in our experimental conditions. Although the signalling components that participate in the regulation of QPRT gene activation are currently unknown, it is of interest that IFN-γ can activate the JAK-STAT or alternative STAT-1-independent signalling pathways which could thus account for a number of discrepancies observed between cell types and species (for review see [31]).

Additionally, a prominent and novel species specific role for the type I interferon IFN-β in the regulation of the entire KP enzymatic machinery in MSCs was demonstrated in our study. Although IFN-β-induced IDO expression has been demonstrated in several cell types including macrophages, dendritic cells and fibroblasts (for review [32], we report that IDO1 gene expression was significantly up-regulated by IFN-β in human MSCs whereas an inhibitory effect was observed in mouse cells. These results have immediate relevance to the study of the pathogenetic role of the KP in diseases when using animal models. Furthermore, the observations shed light on the mechanisms responsible for the immunosuppressive properties of MSCs and IFN-β therapeutic efficacy in autoimmune diseases such as MS [33], [34]. Indeed, although MSC transplantation and IDO induction have been shown to down-modulate neuroinflammation in animal models of MS [20], [26], [35], the impact and functional consequences of KP activation in patients receiving IFN-β treatment and potential stem cell transplants have not been fully explored [36], [37].

Our findings extend evidence that IFN-γ has a significant anti-proliferative effect, which has been previously demonstrated mainly in carcinoma cells (for review see [31]). Although IFNs have been broadly used as anti-tumor, anti-viral and immunomodulatory agents, the precise mechanisms that result in the numerous therapeutic benefits of IFN treatment remain uncertain [31]. Limited work on the effects of IFN-γ on the precursor/progenitor cell growth [38], [39] is expanded here by the identification of an important role of IDO in stem cell proliferation. Together with other biochemical pathways induced by IFN-γ, we show that IDO can inhibit MSC growth *via* depletion of tryptophan, an essential amino acid required for the biosynthesis of proteins. Additionally, the production of downstream tryptophan metabolites (e.g. kynurenine) by MSCs could potentiate and exacerbate the suppressive effects on cell proliferation in an autocrine manner. Although this type of negative feedback has not been demonstrated in MSCs so far, many lines of evidence indicate that kynurenine, hydroxy-anthranilic acid and QUIN have significant inhibitory effects on the proliferation of various cell types [15], [16], [30] [40]. Evidence for these complex mechanisms *in vivo* is still extremely limited, due to the metabolic adaptation processes that usually occur (for review [41], for example, when tryptophan starvation is counteracted by alternative enzymes namely tryptophanyl-tRNA synthase (WRS or TTS) that can maintain an intracellular reservoir of tryptophan available for protein synthesis [27], [28]. WRS can be co-induced by IFN-γ with IDO in various cell types [27], [28], including human and mouse MSCs (***Fig. S4C***, ***Table S2***), however the dynamic interplay between IDO and WRS is not completely understood. It is notable that our findings show that despite the presence of WRS in MSCs and the subsequent short-term counterbalance of the tryptophan depletion, this was not sufficient to compensate the impairment of cell proliferation associated with long-term IFN-γ-induced IDO activation.

Indeed, there are multiple mechanisms of induction and regulation associated with the activation of IDO (for review, see [42]). First, IDO activity can deplete tryptophan in local microenvironment and can also generate downstream KP products but neither mechanism alone can explain the diversity of IDO-mediated effects [42]. In particular, combined effects of tryptophan depletion and presence of KP metabolites are required for IDO-mediated inhibition of T-cell proliferation *in vitro* [43] and for the conversion of naive CD4+CD25- T cells into CD4+CD25+FOXP3+ Treg cells [44].

Second, as discussed above, WRS activity could be another prerequisite for IDO-mediated effects. We have shown that IFN-γ has been involved in WRS upregulation in MSCs, most likely limiting tryptophan availability for IDO and reversing the IDO-mediated tryptophan depletion. It is presently believed that the balance between WRS and IDO activity will dictate tryptophan availability for protein synthesis or for breakdown into KP metabolites [45].

Thirdly, tryptophan availability is also regulated *via* amino-acid transport systems across the membranes of mammalian cells named LAT transporters, which can exchange tryptophan and kynurenines in a bidirectional process (for review, see [45]). Recent evidence for a tryptophan influx/kynurenine efflux cycle suggests that intracellularly produced kynurenines may serve as substrates for the exchange of extracellular tryptophan by LAT transporters [46] and most likely represents a rate-limiting step for IDO-mediated tryptophan degradation (for review, see [45]). In this respect, the literature indicates that in addition to acting at the IDO enzyme active site, the IDO inhibitor 1MT inhibits the LAT system in a dose dependent manner and thus limits tryptophan availability [45], [47], [48]. Therefore, the mechanistic basis for IDO inhibitors action remains to be thoroughly investigated [45].

Finally, IDO-mediated tryptophan depletion and production of KP metabolites can activate GCN2 kinase, and this pathway is not inhibited by the addition of exogenous tryptophan [40], [44], [45], [49]. It should be also pointed out that recent data provide direct evidence supporting the existence of a second substrate binding site in human IDO [50]. Steady-state kinetic data showed that, at high concentrations, tryptophan can bind to the inhibitory substrate binding site of human IDO in addition to the active site, thereby accounting for the substrate inhibition behaviour of the enzyme [50].

To our knowledge this is the first study to link IDO to the control of the differentiation potential of stem cells. Specific IFN-γ- and IFN-β-induced IDO activation in mouse and human MSCs caused significant changes at the transcriptional and protein level of neural, adipocytic and osteocytic markers after differentiation induction procedures. Although previous studies have indicated that type I and type II interferons can affect precursor cell proliferation and differentiation and consequently can influence the potential for tissue repair [38], [39], [51], [52], [53], no definitive mechanism has been identified until now. IFN-γ-induced IDO activity has classically been linked to anti-proliferative effects on immune cells including cytotoxic T cells, leading to the induction of immune tolerance during infection, pregnancy, transplantation, autoimmunity and cancer (see [54] for review). From our study, it is apparent that the KP expression in stem cells plays an additional key role in the control of cell proliferation and differentiation. As such, the manipulation of IDO activity in MSCs and NSCs and the *in vivo* administration of KP inhibitors or synthetic tryptophan metabolites may represent an attractive strategy in a variety of clinical settings, including neurological disorders [26]. In support of this concept, it has been shown that transplantation of MSCs and NSCs can contribute to tissue repair and functional recovery not only though their transdifferentiation into neural cells, but equally through the release of immunoregulatory factors (i.e. KP metabolites), trophic support and possibly the control of proliferation and differentiation of endogenous stem/progenitor cells [55]. Lastly, our findings raise the possibility that the KP machinery might be present and functional in other stem cell types (***Table 1***, ***Fig. 2D***), which together with emergent *in vivo* findings [56] point towards a direct molecular link between tryptophan metabolism and adult neurogenesis, thus offering entirely novel therapeutic opportunities.

## Materials and Methods

### Culture of human Mesenchymal Stromal Cells (hMSCs)

Human MSC were from the kind gift of Professor Darwin J. Prockop, Centre for Gene Therapy, Tulane University, New Orleans, LA, USA. The cells were prepared from bone marrow aspirates (20 mL) taken from the iliac crest of healthy adult donors after informed consent as described previously by Sekiya *et al.* [57]. Purified MSCs were negative for hematopoietic markers CD11b, CD14, CD34, CD45, CD117, HLA-DR, positive for CD105, CD73 and CD90 (data not shown) and consistently differentiated *in vitro* into adipocytes, osteocytes and chondrocytes. To expand a culture, a frozen vial of MSCs (1 million cells, passage 1 or 2) was thawed, resuspended in alpha minimal essential medium (aMEM; Gibco®, Invitrogen, Australia), 20% ES (Embryonic stem cell qualified fetal bovine serum (ESFBS, Invitrogen), 100 U/mL penicillin, 100 µg/mL streptomycin and 2 mM L-glutamine (Gibco, Invitrogen), plated in a 75 cm^2^ culture flask (Nunc), and incubated at 37°C in 5% CO_2_ for further passages until sufficient expansion. Five different experiments were performed with MSCs from three different healthy donors and representative results are presented.

### Culture of mouse Mesenchymal Stromal Cells (mMSCs)

All animals (C57BL/6J mice) were handled in strict accordance with institutional guidelines for animal care approved by the Animal Care and Ethics Committees (ACEC), University of Sydney and UNSW, Sydney, NSW, Australia (approval number N08/32). Mouse MSCs were prepared from bone marrow as previously described [29], [57]. Briefly, C57BL/6J (B6) mice were anesthetized with Avertin (2, 2, 2-Tribromoethanol) and killed by cervical dislocation. Mouse femurs and tibias were aseptically dissected and bone marrow was extruded by flushing media used for the culture using a 10-mL syringe and a 21-gauge needle. The cell suspension obtained was centrifuged at 500 g for 10 minutes and washed with fresh medium: α-MEM or DMEM/F12 (Gibco, Invitrogen) containing 10% non-inactivated ESFBS (Gibco, Invitrogen), specially tested for the ability to sustain the growth of MSCs, 2 mmol/L L-glutamine (JRH Bioscience), 100 units/mL penicillin and 100 mg/mL streptomycin (penicillin-streptomycin, GSL) [58]. The cell suspension was passed through a 70 µm nylon filter and centrifuged at 500 g for 10 minutes, washed one more time and centrifuged at 500 g for 10 minutes. In order to remove the erythrocytes, the cell suspension was treated with lysis buffer (0.144 M ammonium chloride, 17 mM TrisHCl). Viable cells were counted after a trypan blue stain (Sigma) using a Neubauer hemocytometer. Cell suspension at a density of 20×10^6^ whole marrow cells per mL media was either: i) sorted using MACS microbeads; ii) plated in chamber slides (SlideFlask, 10 cm^2^, Nunc) for immunostaining; iii) plated in 6-well plates for selection of mMSCs by adhesion to plastic dishes and subcultures, for RNA isolation and differentiation assessments. When the mMSCs became confluent, they were resuspended with 0.25% trypsin (JRH Biosciences, Brooklyn, Australia) and then subcultured. The media was changed twice a week and the cells cultured in humidified 5% CO_2_/95% air (37°C). Custom made tryptophan-free DMEM/F12 media (Gibco, Invitrogen) was used for the proliferation experiments. Five different experiments were performed with different C57BL/6J mice and representative results are presented.

### Cell selection using magnetic cell sorting (MACS method)

Fresh murine bone marrow cells and mMSC cultures obtained by classic plastic adherent selection were immunodepleted using the following MACS microbeads (Miltenyi Biotec) [29]: lineage cell depletion kit (mature hematopoietic cells, T cells, B cells, monocytes/macrophages, granulocytes and erythrocytes), Ter119 (erythrocytes and erythroid precursor cells), CD45 (cells of hematopoietic origin), CD11b (myeloid cells), c-kit/CD117 (hematopoietic progenitor cells, myeloid, erythoid and lymphoid precursor cells, few mature hematopoietic cells), Sca-1 (hematopoietic cells with differentiation potential) according manufacturer's instructions. Briefly, the magnetically labelled lineage positive cells are depleted by retaining them on a MACS® column (Miltenyi Biotec) in the magnetic field of a MACS Separator, while the unlabelled lineage negative cells pass through the column. Both fractions were resuspended in medium and placed in chambers slides at a concentration of 1×10^6^ cells per mL.

### Modulation of kynurenine pathway expression in MSCs – cytokine and IDO inhibitor treatment

MSC cultures were seeded into T75 culture flasks for protein analysis by western-blot, into 12-well plate for mRNA analysis (triplicate) and into 9 cm^2^ slideflask (Nunc) for immunocytochemistry. Subconfluent cultures were treated separately with 100 IU/ml of recombinant TNF-α and IFN-g (Biosource, Camarillo, CA, USA). In addition, MSCs were treated with different doses of human IFN beta-1b (100, 1,000 and 2,000 IU/ml) (Betaferon®, Schering Pty. Ltd), commercially available mouse IFN-β (Biosource), and mouse IFN-β kindly donated by Biogen Idec. In each case, RNAs and protein samples were collected after 72 hours of incubation and stored at −70°C until use. Inhibition of IDO activity in mouse and human MSCs was performed with norharmane (15 µM), D- and L-isomers of 1-methyl tryptophan (100 µM) (Sigma).

Mouse and human MSCs were tested at different passages (spanning from P4 to 51) for each type of experiment to ensure the reproducibility of our results, even at late passages. Results were consistent at all passages.

### Isolation and culture of mouse NSCs

Mouse mNSCs were purchased from StemCell Technologies and cultured in the NeuroCult®NSC proliferation supplemented with 20 ng/ml of EGF (Biosource) or the differentiation media provided by the manufacturer. Because mNSCs proliferate in neurosphere forms, they were passaged every three days using the NeuroCult Chemical dissociation kit (StemCell Technologies) or mechanical trituration to dissociate them into single cells.

### Measurement of cell viability

AlamarBlue® testing was conducted according to the manufacturer instructions (Biosource). Briefly, after incubation of MSCs for a period of 3 hours with 1/10th volume of AlamarBlue® in the culture medium, the colour change in the supernatants corresponding to the metabolised AlamarBlue® was measured at an OD of 620 nm. The reduction of AlamarBlue® is an indirect measure of cell numbers and produces linear results with a high specificity and sensitivity. Cell numbers for each experimental condition was calculated by linear regression in relation to a standard curve derived from controls of untreated cells plated at different densities.

### Quantitative real-time RT-PCR (qRT-PCR) analysis

qRT-PCR was optimised and performed according to MIQE guidelines [59]. Total RNA was isolated from MSCs, human and mouse cells and tissues by the guanidium-thyocyanate method using Trizol (Gibco, Invitrogen) followed by phenol extraction. After quantification of nucleic acids by spectrophotometry (Nanodrop, Thermo Scientific), RNA samples (2 µg) were subjected to reverse transcription using AMV Reverse Transcriptase (Roche) according to the supplier's instructions. cDNA products were amplified on a Lightcycler® 480 PCR system (Roche Diagnostics) in 20 µl of reaction mixture containing the SYBR GreenER™ qPCR SuperMix Universal (Invitrogen) and 50 µM of forward and reverse primers (***Table S1***). A standard curve with five dilutions steps and three replicates at each dilution steps was constructed and overall amplification efficiency was calculated from its slope as E = 10^−1/slope^−1. All primers have similar amplification efficiency (data not shown but can be provided on request). Each amplification cycle consisted of an initial step at 95°C (5 minutes), followed by 45 cycles of denaturation at 95°C (10 seconds), annealing at 60°C (15 seconds), and extension at 72°C (15 seconds). Quantification of the levels of gene expression for each sample was calculated using the comparative C_q_ method (ΔΔC_q_). Results are expressed relative to the reference genes GAPDH or β-actin. To confirm product specificity, a melting curve analysis was performed after each amplification.

### Immunocytochemistry

The cells were rinsed with PBS (JRH), fixed with 4% paraformaldehyde (ProSciTech) at room temperature for 20 minutes and washed twice in PBS. Cells were then permeabilized with 0.1% Triton X-100 in PBS for 10 minutes. Following one wash in PBS, the cells were treated with 10% normal goat serum (NGS) (Sigma) at room temperature during one hour and washed 3 times in PBS. Cells were then incubated with primary antibodies (diluted in 5% NGS in PBS) at room temperature during 2 hours. Monoclonal antibody directed against IDO was a kind gift from Prof Osamu Takikawa (Japan). Anti-MAP2, -O1, -O4, -A2B5, -Oct4, -NSE, -Olig1, -nestin (Chemicon, Australia) and anti-GFAP (Novocastra, Australia) antibodies were used according to manufacturer's instructions. Monoclonal IgG1, IgG2a and IgM negative controls were used in the same conditions. After three washes in PBS, the cells were incubated with secondary antibodies (diluted in 5% NGS in PBS) for 1 hour in the dark (Alexa Fluor® 488 and Alexa Fluor® 488 594 goat anti-mouse IgG or IgM, Molecular Probes, Invitrogen). After 3 washes in PBS, cells were treated 5 minutes with 4′,6-diamidino-2-phenylindole dihydrochloride (DAPI, Sigma) diluted to 1/500 in water. After three washes in PBS, slides were then mounted with Fluoromount-G (SouthernBiotech) and a coverslip sealed to the slide. The slides were examined with a fluorescence microscope (Olympus BX61) and quantification performed using the Image Pro-Plus 5.0 software (Media Cybernetics, Bethesda MD, USA).

### Western-blot

IDO and β-actin expression was determined by western blot analysis using monoclonal antibodies - mouse IgG3 anti-IDO1 (Chemicon) and mouse IgG2a anti-actin (Sigma). Macrophage and MSC cultures were washed with PBS and scraped off into lysis buffer (CelLytic M, Sigma, with cocktail anti-protease, Roche Diagnostics). Protein samples (2.5 to 20 µg) were separated by 12% SDS-PAGE and transferred to a Immobilon-P membrane (Millipore). The blots were blocked in Tris-buffered saline-0.1% Tween-20 containing 5% nonfat milk and then incubated with the primary monoclonal antibodies at a dilution of 1∶500 for 3 hours at room temperature. This was followed by incubation with the appropriate peroxidase-conjugated secondary antibodies (Bio-Rad, Hercules, CA, USA) and ECL detection (Amersham Pharmacia Biotech).

### HPLC

Tryptophan, kynurenine and kynurenic acid were measured as previously described [60], [61]. Briefly, samples were precipitated with an equal volume of 5% trichloroacetic acid, centrifuged at 3000 *g* for 5 mins, and the supernatants used for the analysis. Tryptophan and kynurenine were measured by HPLC using UV absorption at 278 and 363 nm, respectively, by separate detectors arranged in series after the column [60]. The samples were passed though a Waters Bondapak C18 column in a mobile phase consisting of 80 mM sodium acetate, 80 mM citric acid, pH 5, and 5% acetonitrile at a flow rate of 1 ml/min. Kynurenic acid was measured with a fluorescence detector using an excitation wavelength of 344 nm and an emission wavelength of 398 nm [61], after passing through a Polymer Laboratories PLRP-5 15×0.46 cm column in a mobile phase consisting of 50 mM sodium acetate and 0.25 M zinc acetate, pH 6.3 to which acetonitrile was added (5%).

### Induction of osteogenic and adipogenic differentiation of MSCs

Osteoblastic differentiation was induced by culturing confluent human and mouse MSCs for two weeks in complete medium supplemented with dexamethasone 10^−7^ M, β-glycerophosphate 10 mM, and acid ascorbic 60 µM (all from Sigma) [29]. Cells were maintained in induction medium, which was changed every two days to overcome the instability of ascorbic acid in neutral pH. After 2 to 3 weeks, cells were fixed for 20 minutes with 4% paraformaldehyde, rinsed twice with PBS and stained with Alizarin red, pH 4.1 for 20 minutes at room temperature. Alternatively, treated cells were used for RNA extraction and qRT-PCR analysis of *IBSP (integrin binding sialoprotein)* and *osteopontin/secreted phosphoprotein 1 (SPP1)* gene expression.

To induce adipocytic differentiation, sub-confluent MSCs cultures were cultured from 1 to 3 weeks in complete medium supplemented with dexamethasone 10^−7^ M, indomethacin 60 µM, insulin 5 µg/ml, IBMX (3-isobutyl-1-methylxanthine) 0.5 mM, and hydrocortisone 0.5 µM [29]. Cells were used for RNA extraction and qRT-PCR analysis of *Fabp4 (fatty acid binding protein 4)*, *adipsin* and *adipoQ* gene expression. Treated cells were fixed with 4% paraformaldehyde and stain with 0.5% Oil Red O in methanol for 20 minutes.

### Induction of neural differentiation of MSCs

Neural differentiation of human and mouse MSCs was performed using a modified two-step protocol from Song *et al*, 2007 [62]. Briefly, for the generation of nestin-positive cells from MSCs, cells were cultured in the presence of nestin induction medium: DMEM/F12 medium, 2% FBS (Gibco, Invitrogen), supplemented with N2 supplement (Invitrogen), 5 µg/ml insulin (Sigma), transferrin 100 µg/ml, 60 µM putrescine, 0.02 µM progesterone, 0.03 µM selenium, together with 20 ng/ml epidermal growth factor (EGF) and 16 ng/ml basic fibroblast growth factor (bFGF, Biosource). After 14 days, the cells continued to proliferate and were passaged when 70–80% confluent. At this stage, the cultures expressed a high proportion (80%) of nestin positive cells. For the neural differentiation of nestin positive cells, cells were replated onto poly-D-lysine-coated cell culture slides and cultured in neural differentiation medium: DMEM/F12 (Gibco, Invitrogen) with N2 supplement (Invitrogen), 20 µg/ml insulin (Sigma), transferrin 100 µg/ml, 60 µM putrescine, 0.02 µM progesterone, 0.03 µM selenium, supplemented with 0.5 µM all-*trans* retinoic acid (Sigma), 10 ng/ml brain derived neurotrophic factor (BDNF, Invitrogen) and 2% FBS, in the presence of 100 IU/ml IFN-γ and/or IDO inhibitors norharmane (15 µM), D-1-methyl-tryptophan (100 µM) and L-1-methyl-tryptophan (100 µM). After 7 days, neuro-MSCs changed morphology and expressed markers of neurons (MAP2, NSE), astrocytes (GFAP) and oligodendrocyte progenitor cells (Olig1, A2B5, O1 and O4) (***Fig. S5***).

### Statistical analysis

Statistical analysis was carried out with GraphPad Prism version 5.0 (GradPad Sofware Inc., San Diego, CA, USA). Differences between two groups were analyzed by the two-tailed Student's *t*-test and of more than two groups by one-way ANOVA with *post-hoc* Dunnett's or Tukey's Multiple Comparison tests. p<0.05 was considered statistically significant. If not differentially indicated, data are representative from at least two independent experiments.

## Supporting Information

Figure S1 (0.10 MB PDF)

Figure S2 (0.37 MB PDF)

Figure S3 (0.42 MB PDF)

Figure S4 (0.71 MB PDF)

Figure S5 (3.48 MB PDF)

Table S1 (0.06 MB PDF)

Table S2 (0.09 MB PDF)

Table S3 (0.07 MB PDF)

Table S4 (0.08 MB PDF)
