# Supplementary material for: Interferon-γ Regulates the Proliferation and Differentiation of Mesenchymal Stem Cells via Activation of Indoleamine 2,3 Dioxygenase (IDO)
Source: PLoS One. 2011 Feb 16;6(2):e14698. doi: 10.1371/journal.pone.0014698 (PMC3040184; doi:10.1371/journal.pone.0014698)
Supplement: Figure S1 — (0.10 MB PDF) [file pone.0014698.s001.pdf]

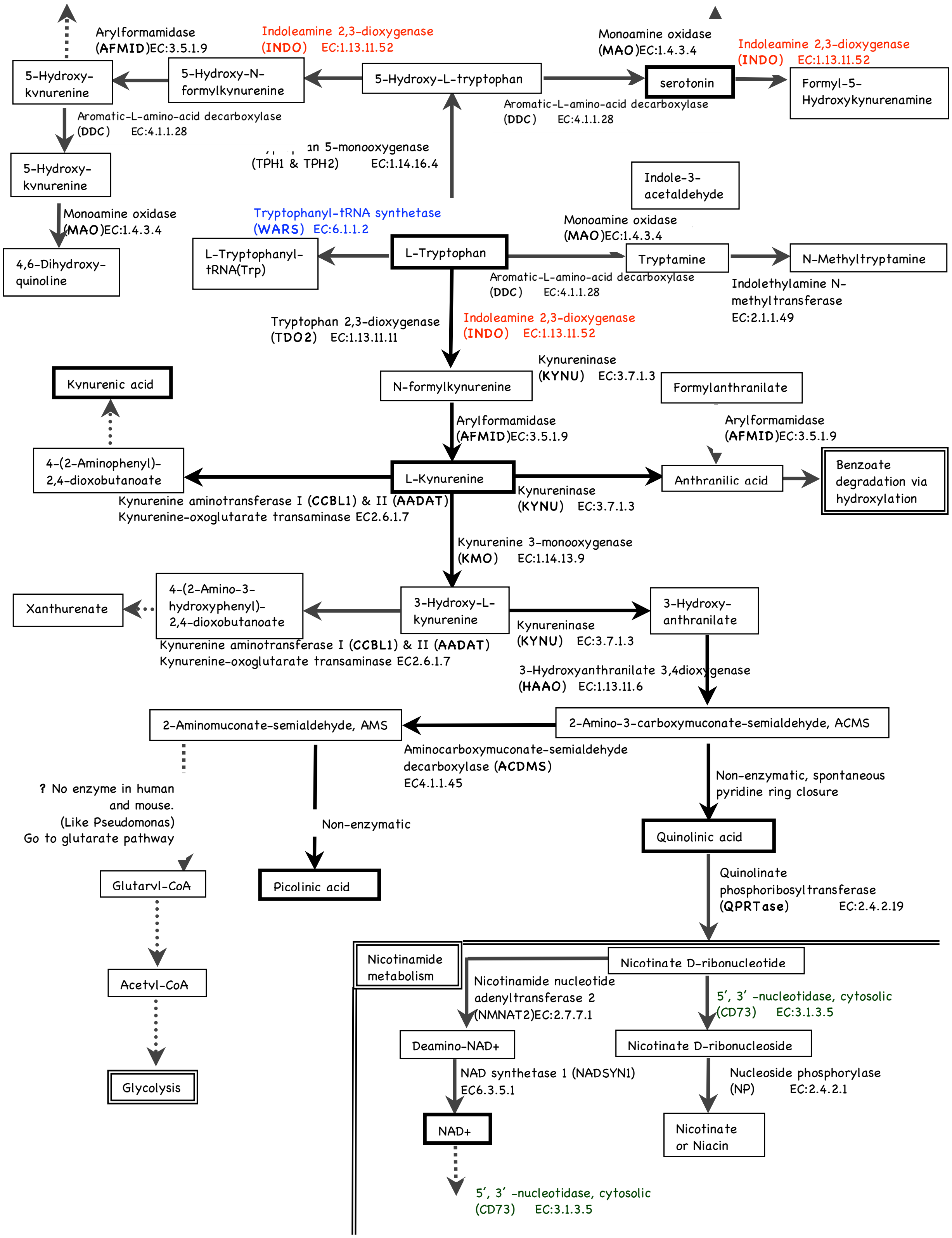

Figure S1: Overview of the kynurenine pathway of tryptophan metabolism (modified from http://www.genome.jp/dbget-bin/get_pathway?org_name=map&mapno=00380)
