# Supplementary material for: Interferon-γ Regulates the Proliferation and Differentiation of Mesenchymal Stem Cells via Activation of Indoleamine 2,3 Dioxygenase (IDO)
Source: PLoS One. 2011 Feb 16;6(2):e14698. doi: 10.1371/journal.pone.0014698 (PMC3040184; doi:10.1371/journal.pone.0014698)
Supplement: Figure S2 — (0.37 MB PDF) [file pone.0014698.s002.pdf]

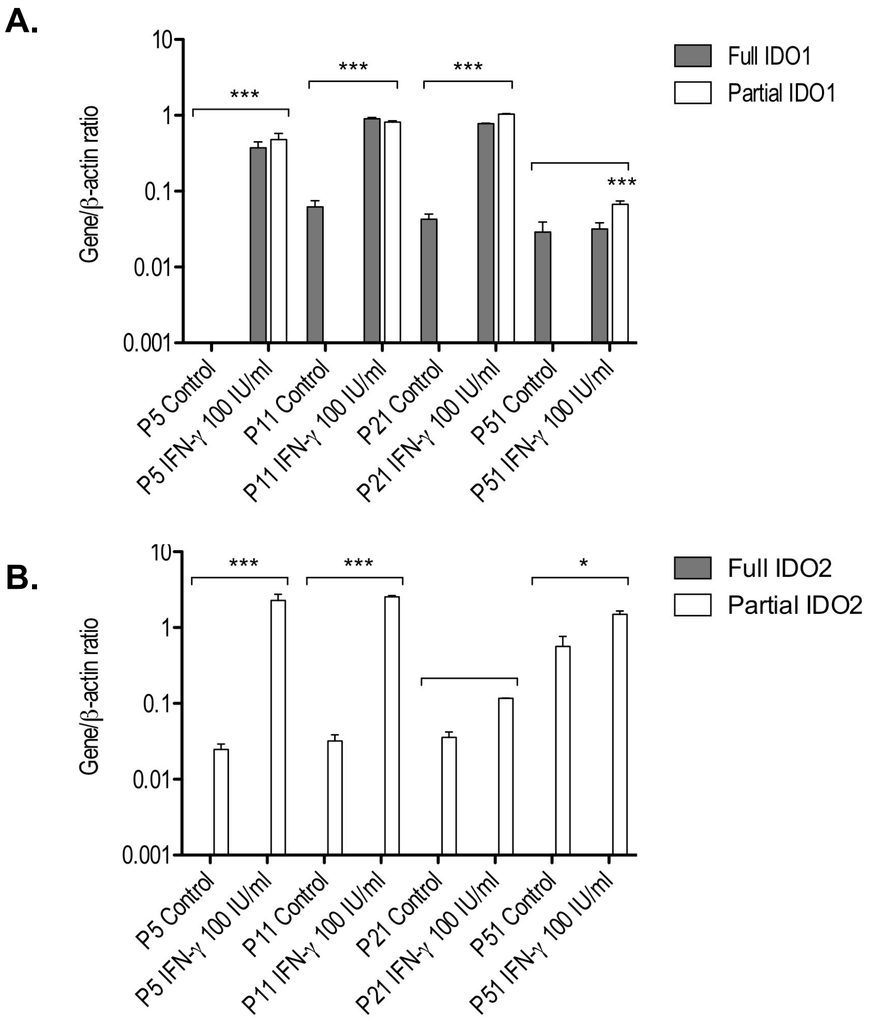

Figure S2: Expression of full and partial IDO1 and IDO2 in mouse MSCs at different passages in culture. Mouse MSCs at different passages were cultured in the absence or presence of IFN-γ (100 IU/ml) or IFN-β (100 IU/ml) for 72 hours. **A:** Expression of full and partial IDO1 in mouse MSCs at passages 5, 11, 21 and 51. **B:** Expression of full and partial IDO2 in mouse MSCs at passages 5, 11, 21 and 51. **C:** IFN-β effect on IDO expression in mouse MSC cultures at passages 5, 10, 20 and 46. The ratios gene/β-actin were multiplied by 10,000 for clarity purposes in A. and B. Data are mean ± standard error (SEM). $^*p<0.05$, $^{**}p<0.01$, $^{***}p<0.001$ when compared with control (without cytokine treatment). Differences between experimental groups were analyzed by one-way ANOVA with *post-hoc* Tukey's Multiple Comparison tests. Abbreviations: IFN-γ, interferon-γ; IFN-β, interferon-β; MSCs, mesenchymal stem cells; IDO1 and IDO2, indoleamine 2,3-dioxygenase 1 and 2.
