# Supplementary material for: Interferon-γ Regulates the Proliferation and Differentiation of Mesenchymal Stem Cells via Activation of Indoleamine 2,3 Dioxygenase (IDO)
Source: PLoS One. 2011 Feb 16;6(2):e14698. doi: 10.1371/journal.pone.0014698 (PMC3040184; doi:10.1371/journal.pone.0014698)
Supplement: Figure S3 — (0.42 MB PDF) [file pone.0014698.s003.pdf]

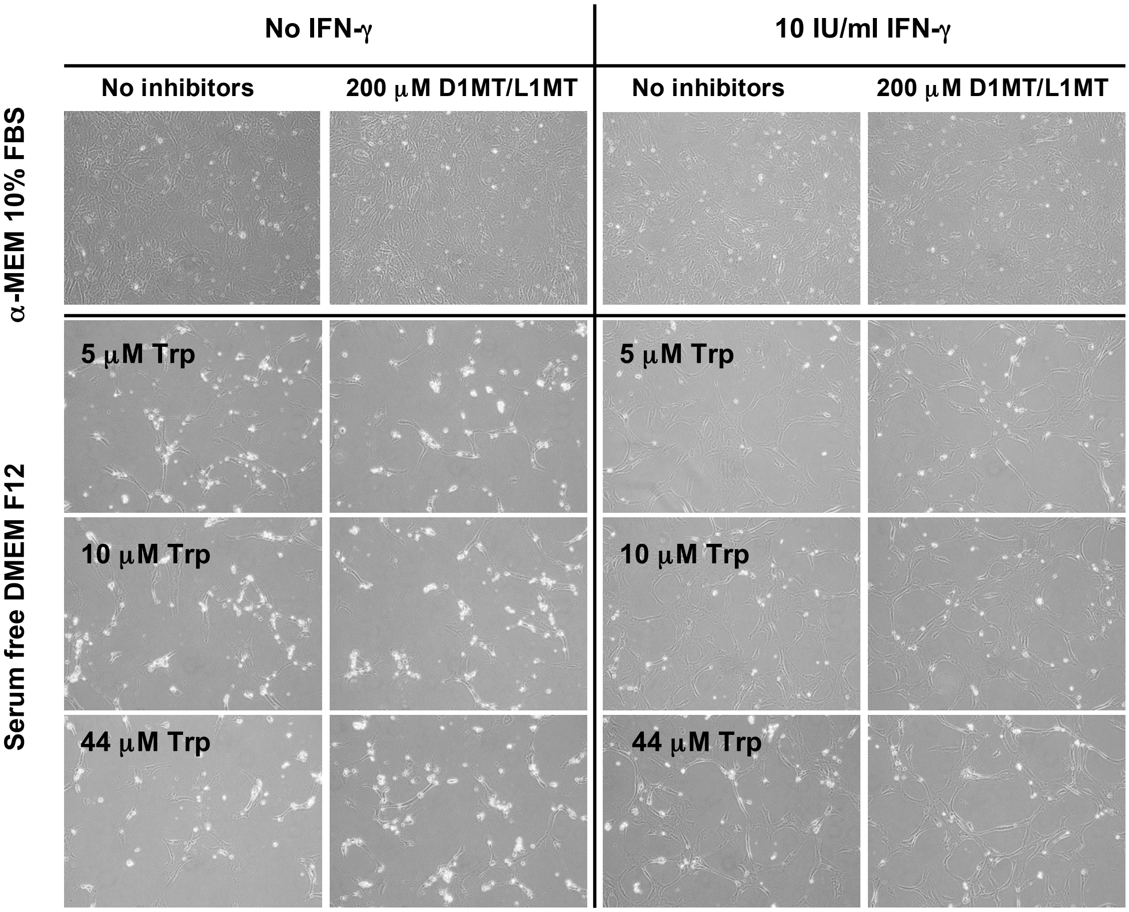

Figure S3: Morphology of mouse MSCs in culture with IFN-γ and IDO inhibitors. Representative phase contrast photographs of mouse MSCs (passage 20) cultured in serum free DMEM F12 medium in the presence of 10 IU/ml IFN-γ, increasing concentrations of tryptophan (0, 5, 10 and 44 μM) and/or IDO inhibitors D-1-methyl-tryptophan and L-1-methyl-tryptophan (100 μM) for 6 days. Mouse MSCs were cultured with 10% FBS as positive controls. Abbreviations: IFN-γ, interferon-γ; MSCs, mesenchymal stem cells; IDO, indoleamine 2,3-dioxygenase; Trp, tryptophan; D-1MT, D-1-methyl-tryptophan; L-1MT, L-1-methyl-tryptophan; FBS, foetal bovine serum; F12, DMEMF12. Magnification X100.
