# Supplementary material for: Interferon-γ Regulates the Proliferation and Differentiation of Mesenchymal Stem Cells via Activation of Indoleamine 2,3 Dioxygenase (IDO)
Source: PLoS One. 2011 Feb 16;6(2):e14698. doi: 10.1371/journal.pone.0014698 (PMC3040184; doi:10.1371/journal.pone.0014698)
Supplement: Figure S4 — (0.71 MB PDF) [file pone.0014698.s004.pdf]

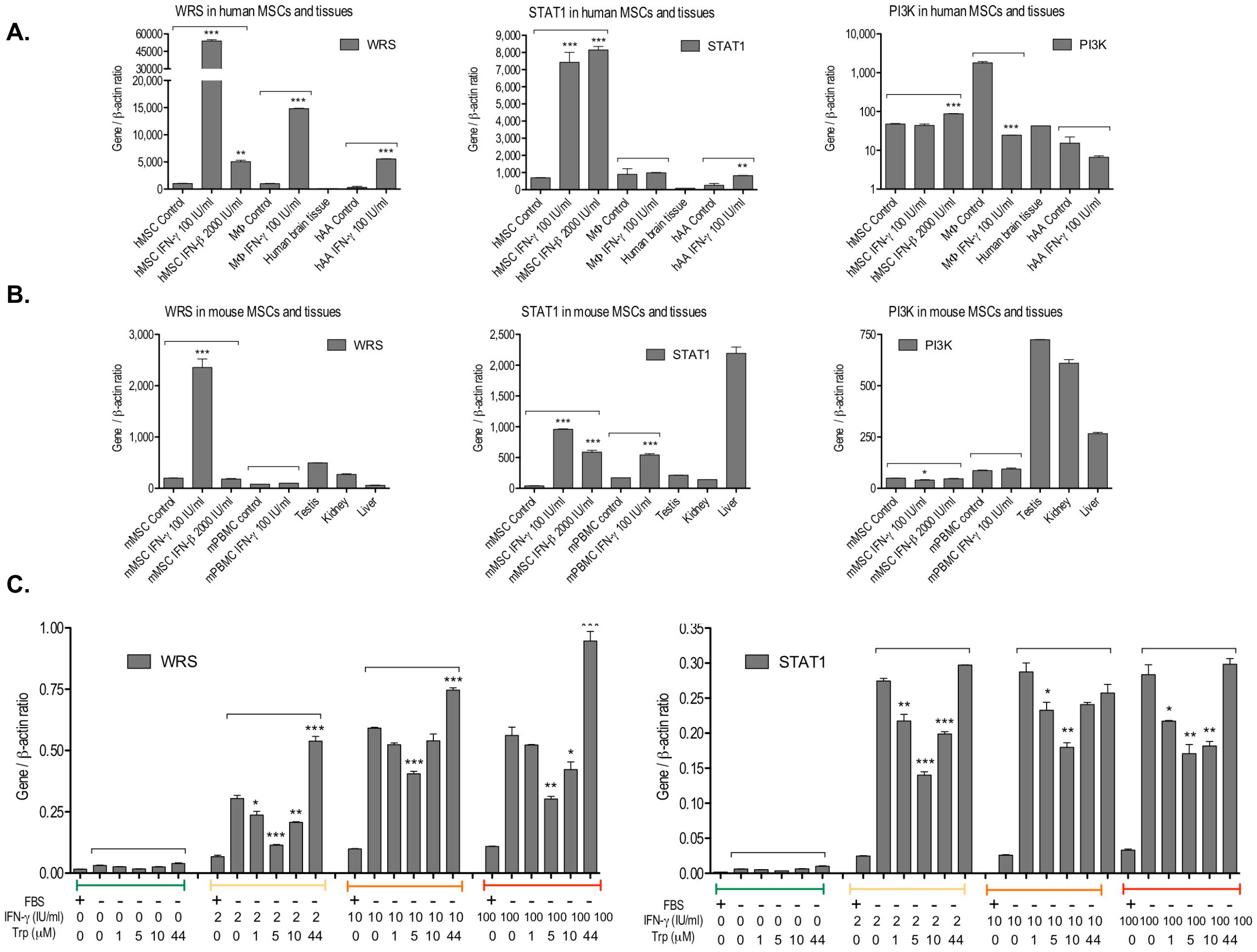

Figure S4: WRS, STAT1 and PIK3 expression in human and mouse tissues and MSC cultures. Human and mouse MSCs were cultured in the absence or presence of IFN-γ (2, 10 or 100 IU/ml) or IFN-β (2,000 IU/ml) for 72 hours. Data are mean ± standard error (SEM). $^{*}p<0.05$, $^{**}p<0.01$, $^{***}p<0.001$ when compared with control (no treatment). Differences between two groups were analyzed by the two-tailed Student's *t*-test and of more than two groups by one-way ANOVA with *post-hoc* Dunnett's and Tukey's Multiple Comparison test. The gene/β-actin ratios were multiplied by 10,000 for clarity purposes in A. and B. **A.** Gene expression of WRS, STAT1 and PI3K in human tissues and MSC cultures as measured by qRT-PCR. **B.** Gene expression of WRS, STAT1 and PI3K in mouse tissues and MSC cultures as measured by qRT-PCR. **C.** Expression of WRS and STAT1 mRNA in mouse MSCs as measured by qRT-PCR. Cells were grown in the presence of increasing concentrations of tryptophan (0, 1, 5, 10 and 44 μM) and/or IFN-γ (0, 2, 10 and 100 IU/ml) for 24 hours. Mouse MSCs were cultured with 10% FBS as positive controls. Abbreviations: IFN-γ, interferon-γ; IFN-β, interferon-β; MSCs, mesenchymal stem cells; hAA, human adult astrocytes; MΦ, macrophages; Trp, tryptophan; KYN, kynurenine; KYNA; kynurenic acid; FBS, foetal bovine serum; STAT1, signal transducer and activator of transcription 1; PI3K, phosphoinositide 3-kinase; WRS, tryptophanyl-tRNA synthase.
