# Supplementary material for: Interferon-γ Regulates the Proliferation and Differentiation of Mesenchymal Stem Cells via Activation of Indoleamine 2,3 Dioxygenase (IDO)
Source: PLoS One. 2011 Feb 16;6(2):e14698. doi: 10.1371/journal.pone.0014698 (PMC3040184; doi:10.1371/journal.pone.0014698)
Supplement: Figure S5 — (3.48 MB PDF) [file pone.0014698.s005.pdf]

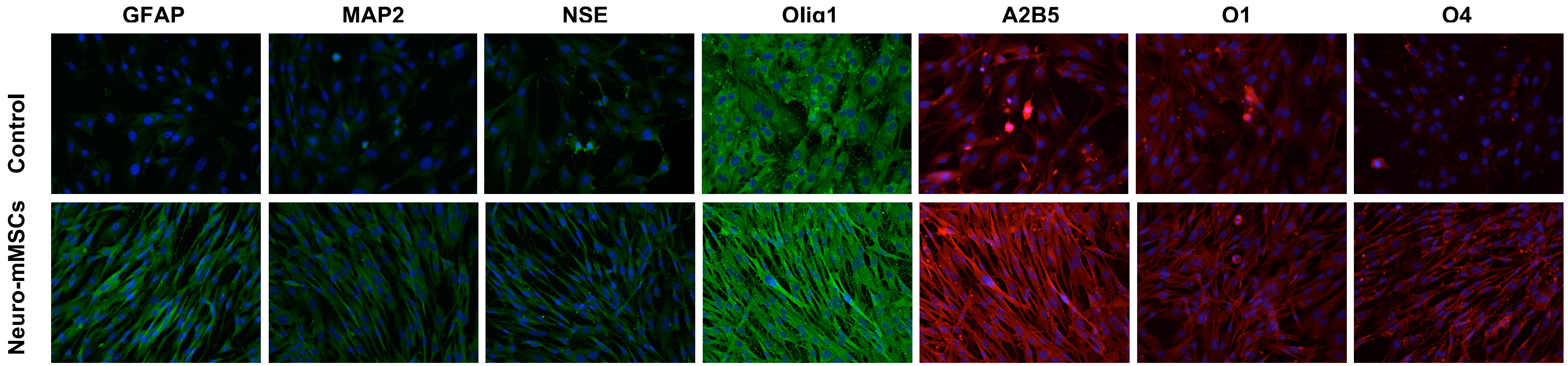

Figure S5: Expression of neural markers by mouse and human MSCs after neural differentiation procedures. Representative photographs of fluorescent immunocytochemical labelling of differentiating mouse MSCs cultured for 14 days using the nestin induction medium then 7 days in neural differentiation media as described in *Material and Methods.* DAPI-stained nuclei are shown in blue. Images were taken under identical exposure conditions (Magnification X200). Abbreviations: GFAP, glial fibrillary acidic protein; MAP2, microtubule-associated protein 2; NSE, neuron-specific enolase.
