# Supplementary material for: Interferon-γ Regulates the Proliferation and Differentiation of Mesenchymal Stem Cells via Activation of Indoleamine 2,3 Dioxygenase (IDO)
Source: PLoS One. 2011 Feb 16;6(2):e14698. doi: 10.1371/journal.pone.0014698 (PMC3040184; doi:10.1371/journal.pone.0014698)
Supplement: Table S1 — (0.06 MB PDF) [file pone.0014698.s006.pdf]

TABLE S1: Primers for quantitative real-time RT-PCR

HUMAN PRIMERS	FORWARD PRIMER	REVERSE PRIMER	SIZE	REFERENCE
**h b-ACTIN**	CATGTACGTTGCTATCCAGGC	CTCCTTAATGTCACGCACGAT	250bp	Primerbank ID 4501885a1
**full h IDO1**	GGCTTTGCTCTGCCAAATCC	TTCTCAACTCTTTCTCGAAGCTG	119bp	PrimerBank ID 4504577a1
**partial p IDO1**	GATGTCCGTAAGGTCTTGCCA	TGCAGTCTCCATCACGAAATG	187bp	PrimerBank ID 4504577a3
**full h IDO2**	AAATGCACTGCCAGTTGAAA	CTGGTGGGTGAAGTGTCAAG	174bp	FL design from NM_194294
**partial h IDO2**	CTGATCACTGCTTAACGGCA	TGCCACCAACTCAACACATT	281bp	FL design from NM_194294
**h TDO2**	GGTTCCTCAGGCTATCACTACC	CAGTGTCGGGGAATCAGGT	101bp	PrimerBank ID 5032165a1
**h KYNU**	ACCTGCATTAGTGGGATGGTT	CAGACCAACAAAATGGGAGGAT	123bp	PrimerBank ID 4504937a1
**h AADAT**	GGCTGGTGGCTTACCAAATC	ACTCGGAGAATACTGAAGTGCTC	124bp	PrimerBank ID 33469970a2
**h KMO**	TGTCAACTCAAGCTGGTTCATT	TGGCTATCAGTGATCCCAAGAAA	194bp	PrimerBank ID 4504891a1
**h ACMSD**	CAGCACCGTTGTGAGCTAC	TGCCGCATAGACAGGAAAGAG	187bp	PrimerBank ID 19911227a2
**h CCBL1**	CAGTAGCCGAGAGCTTTGAAC	CTTCAGGCCCACTGACTGTA	131bp	PrimerBank ID 21707106a3
**h TPH1**	TGGCTGAACCTAGTTTTGCCC	CCAAAGACTCTTAGCTGTCCATC	151bp	PrimerBank ID 4759248a3
**h TPH2**	ATACCTGAGCCCACGAGACTT	CATGTCCCAAGAGTTCATGGC	131bp	PrimerBank ID 31795563a3
**h AFMID**	GCAGAGCGGAAGTAAGGATG	TCCCTTGTTGCTTGGATACC	175bp	FL design primer3 from NM_001010982.2|
**h HAAO**	ACACCGGGATGTGGTCATTC	ATAGTACCTGAGCCCATCTAGC	194bp	FL design primer3 from NM_012205.1|
**h QPRT**	TGTTGAAGGATAACCATGTGGTG	CTGCTGCATTCCACTTCCA	106bp	PrimerBank ID 7657488a1
**h WARS**	ACAAGGGGAGCTCGTAAGGT	TATGCCTTTTGCACTGCTTG	241bp	FL design primer-blast from NM_173701.1
**h WARSII**	ATTACTGTCCCCCAAGACCC	CATCGTGCTTCTGCTTGGTA	223bp	FL design primer-blast from NM_015836.3
**h STAT1**	CTAGTGGAGTGGAAGCGGAG	CACCACAAACGAGCTCTGAA	252bp	FL design primer-blast from NM_007315.3
**h PIK3**	CCCCTCCATCAACTTCTTCA	CGGTTGCCTACTGGTTCAAT	155bp	FL design primer-blast from NM_006218.2
**h CNP1**	CTGCTAGAGTGCAAGACGCTC	CAGCCAGGTCCTCATCGAG	190bp	PrimerBank ID 14916481a2
**h GALC**	GCAACCTCCCGACTTCTAGTA	ACCACTCGTATCCTCGGAAATA	199bp	PrimerBank ID 457446a3
**h GFAP**	CCTCTCCCTGGCTCGAATG	GGAAGCGAACCTTCTCGATGTA	161bp	PrimerBank ID 4503979a1
**h MAP2**	CTGCTTTACAGGGTAGCACAA	TTGAGTATGGCAAACGGTCTG	135bp	PrimerBank ID 24416560a1
**h NESTIN**	GAAACAGCCATAGAGGGCAAA	TGGTTTTCCAGAGTCTTCAGTGA	167bp	PrimerBank ID 35019a2
**h ID2**	GACCCGATGAGCCTGCTATAC	AATAGTGGGATGCGAGTCCAG	165bp	PrimerBank ID 31982933a1
**h NG2**	CACGGCTCTGACCGACATAG	CCCAGCCCTCTACGACAGT	233bp	PrimerBank ID 4503099a1
**h SLC1A3**	ATCCTTGGATTTACCCTCCGA	CGCCATTCCTGTGACAAGAC	141bp	PrimerBank ID 31543628a1
**h HES1**	ATGGAGAAAAATTCCTCGTCCC	TTCAGAGCATCCAAAATCAGTGT	182bp	PrimerBank ID 5031763a1
**h SLC1A1**	GCGAGGAAAGGATGCGAGT	GCTGTGTTCTCGAACCAAGACT	114bp	PrimerBank ID 31543626a1
**h GRM1**	AGACCAATGAGACGGCCTG	CCTCCTCTACGTTGTAAAGGGT	126bp	PrimerBank ID 4504135a1
**h NPDC1**	CGCCCTTGTGCTGATCCTG	TGCTGGTAGTGGTACATCTCC	210bp	PrimerBank ID 20149617a1
**h FABP4**	AGCACCATAACCTTAGATGGGG	CGTGGAAGTGACGCCTTTCA	132bp	PrimerBank ID 4557579a1
**h CFD (Adipsin)**	GACACCATCGACCACGACC	GCCACGTCGCAGAGAGTTC	128bp	PrimerBank ID 4503309a1
**h SPP1 (osteopontin)**	ACTCGAACGACTCTGATGATGT	GTCAGGTCTGCGAAACTTCTTA	224bp	PrimerBank ID 4759166a2
**h IBSP**	GAATGGCCTGTGCTTTCTCAA	TCGGATGAGTCACTACTGCCC	169bp	PrimerBank ID 4826766a1

MOUSE PRIMERS	FORWARD PRIMER	REVERSE PRIMER	SIZE	REFERENCE
**m b-actin**	GGCTGTATTCCCCTCCATCG	CCAGTTGGTAACAATGCCATGT	154bp	PrimerBank ID 6671509a1
**full m ido1**	TGGCACTCAGTAAAATATCTCCT	CAGGCAGATTTCTAGCCACA	153bp	FL design from NM_008324.1
**partial m ido1**	ATTGGTGGAAATCGCAGCTTC	ACAAAGTCACGCATCCTCTTAAA	159bp	PrimerBank ID 6680347a3
**full m ido2** (mINDOL1)	ATGGAGCCTCAAAGTCAGAGC	CGCTGCTCACGGTAACTCTTTA	242bp	H.J. Ball *et al.* Gene 396 (2007) 203–213
**partial m ido2** (mINDOL1)	CCTCATCCCTCCTTCCTTTC	GGAGCAATTGCCTGGTATGT	217bp	FL design primer3 from NM_145949
**m tdo2**	AGGAACATGCTCAAGGTGATAGC	CTGTAGACTCTGGAAGCCTGAT	156bp	PrimerBank ID 31982697a3
**m kynu**	TCAAACCCTCCCATTTTGTTGG	CCCCTTGTTTTCGGTGTTATCTT	162bp	PrimerBank ID 27229113a2
**m aadat**	ATGAATTACTCACGGTTCCTCAC	AACATGCTCGGGTTTGGAGAT	137bp	PrimerBank ID 6754408a1
**m kmo**	TGATGTGTACGAAGCTAGGGA	TCATGGGCACACCTTTGGAAA	146bp	PrimerBank ID 19527030a2
**m acmsd**	TGAATGACCCGGAACTCTTC	ATGTTGAATCCATGGGCAAT	271bp	FL design from NM_001033041.2
**m ccbl1**	CGAAGGCTGGAAGGGATCG	GCGGTGAGAAGTCAGGGAA	106bp	PrimerBank ID 31982063a1
**m tph1**	CATCAGCCGAGAACAGTTGA	TTCGGATCCATACAACAGCA	185bp	FL design from NM_009414.2
**m tph2**	GGGCTGGTGAAAGCACTTAG	GAGGACTCGGTGAGAGCATC	282bp	FL design from NM_173391.2
**m afmid**	TTGGGAACTTCGTGCAGATAGG	CATGAAGGCCGAGTCATCTTTAC	206bp	PrimerBank ID 21746157a2
**m haao**	GGAGGCCCCAATACCAGGA	TATAGGCACGTCCCGGTGTT	120bp	PrimerBank ID 15277547a2
**m qprt**	CCGGGCCTCAATTTTGCATC	GGTGTTAAGAGCCACCCGTT	240bp	PrimerBank ID 19526852a1
**m wars**	CAGGATGTGTTCAATGTGCC	TTGAAGGTGACGTGCTTCTG	233bp	FL design primer-blast from NM_011710.2
**m warsII**	GGAGAGCGAGTGTTTTCTGG	GGAGGTCCACGATGCTGTAT	130bp	FL design primer-blast from NM_027462.4
**m stat1**	TGGTGAAATTGCAAGAGCTG	CAGACTTCCGTTGGTGGATT	158bp	FL design primer-blast from NM_009283.3
**m pik3**	ACTGTTCAGAGAGGCCAGGA	CGGTTGCCTACTGGTTCAAT	180bp	FL design primer-blast from NM_008839.1
**m adipsin**	CATGCTCGGCCCTACATGG	CACAGAGTCGTCATCCGTCAC	129bp	PrimerBank ID 7304867a1
**m adipoQ (adiponectin)**	TGTTCCTCTTAATCCTGCCCA	CCAACCTGCACAAGTTCCCTT	104bp	PrimerBank ID 31982423a1
**m pparg**	GGAAGACCACTCGCATTCCTT	TCGCACTTTGGTATTCTTGGAG	158bp	PrimerBank ID 6755138a2
**m spp1 (osteopontin)**	TCACCATTCGGATGAGTCTG	ACTTGTGGCTCTGATGTTCC	437bp	FL design primer3 from NM_009263.1|
**m ibsp**	CAGGGAGGCAGTGACTCTTC	AGTGTGGAAAGTGTGGCGTT	158bp	PrimerBank ID 6680335a1
