# Supplementary material for: Interferon-γ Regulates the Proliferation and Differentiation of Mesenchymal Stem Cells via Activation of Indoleamine 2,3 Dioxygenase (IDO)
Source: PLoS One. 2011 Feb 16;6(2):e14698. doi: 10.1371/journal.pone.0014698 (PMC3040184; doi:10.1371/journal.pone.0014698)
Supplement: Table S2 — (0.09 MB PDF) [file pone.0014698.s007.pdf]

		hMSCs Control	hMSCs IFN-γ 100 IU/ml	hMSCs IFN-β 2000 IU/ml	MΦ Control	MΦ IFN-γ 100 IU/ml	Human brain tissue	hAA Control	hAA IFN-γ 100 IU/ml	hAA IFN-β 2000 IU/ml	mMSCs Control	mMSCs IFN-γ 100 IU/ml	mMSCs IFN-β 2000 IU/ml	Thymus	Brain	Testis	Kidney	Liver	Spleen	mPBMCs Control	mPBMCs IFN-γ 100 IU/ml	mNSCs Control	mNSCs IFN-γ 100 IU/ml
**Full IDO1**	Mean	0.0290	11,865.0000	57.0000	0.0000	359.5000	0.0000	0.0035	209.5000	4.8950	0.0001	5.8600	0.0000	4.6050	0.0000	2.4150	0.0423	0.0000	0.0371	0.0000	0.0251	0.1584	0.0660
	SD	0.0000	403.0509	0.4243	0.0000	7.7782	0.0000	0.0030	4.9497	0.0212	0.0002	0.3818	0.0000	0.0636	0.0000	0.1061	0.0031	0.0000	0.0145	0.0000	0.0028	0.0102	0.0112
**Partial IDO1**	Mean	0.0012	1,442.5000	2.1250	0.0000	56.5000	0.0070	0.0118	40.7000	0.6955	0.0000	4.7200	0.0634	0.0596	1.1620	10.6500	0.5970	0.1945	0.3125	0.0000	0.0266		
	SD	0.0000	6.3640	0.0071	0.0000	4.6669	0.0023	0.0167	0.0000	0.0049	0.0000	0.1273	0.0266	0.0000	0.3224	0.9192	0.0339	0.2751	0.0106	0.0000	0.0019		
**Full IDO2**	Mean	0.0047	0.3580	0.0395	15.3500	0.0323	6.5000	0.0106	0.0112	0.0193	0.0000	0.0000	0.0000	0.0000	0.0000	0.3190	21.7500	151.5000	0.0000	0.0000	0.0000	0.6134	0.5784
	SD	0.0020	0.0014	0.0011	0.3536	0.0456	0.3253	0.0077	0.0110	0.0107	0.0000	0.0000	0.0000	0.0000	0.0000	0.0127	1.6263	6.3640	0.0000	0.0000	0.0000	0.0370	0.0617
**Partial IDO2**	Mean	0.0073	0.8985	0.0882	44.1000	0.9595	19.3000	0.0141	0.0990		0.2050	8.2150	0.2590	17.9000	3.1700	1.3020	25.2500	344.5000	0.0987	0.1760	0.1865		
	SD	0.0103	0.0672	0.0035	6.9296	0.1421	0.0000	0.0094	0.0085		0.0057	0.1768	0.0552	10.0409	0.2970	0.5629	2.0506	180.3122	0.0016	0.0085	0.0205		
**KYNU**	Mean	0.3750	9.5450	4.1500	399.0000	287.0000	7.6300	0.1700	0.5950	0.5140	0.0001	0.0000	0.0000		0.0012	0.8625	152.0000	3,819.0000		0.6765	3.3150	0.0113	0.0000
	SD	0.0481	0.4455	0.2546	53.7401	8.4853	0.0000	0.0184	0.0424	0.1047	0.0000	0.0000	0.0000		0.0017	0.0870	0.0000	370.5240		0.0134	1.0677	0.0012	0.0000
**ACDMS**	Mean	0.4490	0.1265	0.5365	11.3300	0.3270	14.0500	0.2200	0.0774	0.0980	0.0024	0.0000	0.0037	0.5030	0.0000	3.5350	1,840.0000	109.2000					
	SD	0.0651	0.0007	0.1619	3.6345	0.2263	0.0707	0.0354	0.0313	0.0410	0.0008	0.0000	0.0000	0.0000	0.0000	0.3465	16.9706	28.0014					
**TPH1**	Mean	0.4755	0.4775	1.2200	6.4550	0.1290	0.9685	0.1096	0.9474	0.1395	0.1865	0.1285	0.2225	1.6600	1.5050	3.3600	0.9030	0.0000					
	SD	0.0389	0.0049	0.0566	2.6375	0.0170	0.0346	0.1533	1.2765	0.0276	0.0134	0.0049	0.0304	0.1556	0.0495	0.9334	0.0000	0.0000					
**TPH2**	Mean	0.0089	0.0079	0.0523	0.0031	0.6117	11.9500	0.0055	0.0106	0.0187	0.0746	0.1290	0.0449	0.0000	7.5150	3.0550	0.3030	0.0040				0.1032	4.6638
	SD	0.0038	0.0029	0.0088	0.0002	0.8179	0.3536	0.0012	0.0121	0.0016	0.0023	0.0000	0.0049	0.0000	0.2051	0.4879	0.0693	0.0000				0.0110	2.1664
**KMO**	Mean	0.0187	0.1081	1.4000	10.5300	178.5000	3.0850	0.0401	0.3272	0.3165	0.0355	0.0387	0.0453		5.7200	137.0000	1,635.0000	20,565.0000		0.1780	0.1616	0.0190	0.0000
	SD	0.0258	0.0268	0.1131	1.5132	3.5355	1.2657	0.0252	0.3943	0.0587	0.0087	0.0012	0.0124		1.2587	11.3137	8.4853	49.4975		0.0636	0.0981	0.0020	0.0000
**HAAO**	Mean	0.2610	0.8015	0.8870	2.1500	41.1000	35.7000	4.5650	1.8900	3.5350	0.4500	0.3005	0.3300		7.4600	3.6150	743.5000	3,973.0000		0.5055	0.4910	0.0000	0.0573
	SD	0.0665	0.0346	0.1146	0.0990	0.4243	17.8191	0.1061	0.3111	0.4172	0.0014	0.0092	0.0339		1.7253	0.7283	13.4350	11.3137		0.0276	0.0354	0.0000	0.0266
**CCBL1**	Mean	3.0750	1.6500	4.0700	12.1500	1.7400	45.3500	2.3000	1.0715	1.2350	9.6200	5.4800	7.2950		37.9000	183.5000	320.0000	2,729.0000		4.6600	5.2850	10.0503	39.1429
	SD	0.4596	0.1131	0.3818	0.4950	0.1273	21.9910	0.0283	0.6060	0.1202	0.1273	0.0141	0.2899		5.6569	3.5355	7.0711	82.0244		0.3394	0.1909	0.7898	18.1827
**AFMID**	Mean	0.4990	0.6870	0.8205	0.0016	0.2430	2.1600	0.5405	0.2775	0.4495	7.3350	7.6850	5.8800		0.3325	11.9000	211.5000	478.5000		0.9800	0.8295	0.2441	0.7937
	SD	0.0339	0.0042	0.1520	0.0023	0.0410	0.9334	0.0163	0.0205	0.0856	0.0778	0.3606	0.1414		0.4702	0.7071	7.7782	17.6777		0.0042	0.1421	0.0260	0.3687
**QPRT**	Mean	0.2165	0.0779	0.7325	0.1233	0.9735	623.0000	6.1300	1.6650	7.5500	7.4800	7.8900	6.4350		0.0012	12.0000	236.5000	485.0000		0.8135	0.8105	0.5222	38.2592
	SD	0.0601	0.0131	0.0672	0.1056	0.0233	52.3259	0.7495	0.0071	0.0566	0.0424	0.4950	0.5020		0.0017	0.5657	10.6066	26.8701		0.0714	0.1365	0.0557	17.7723
**AADAT**	Mean	20.0000	18.5000	35.1000	23.3000	0.6275	22.9500	3.2550	1.7550	1.7550	0.2090	0.1710	0.2045		1.0755	0.1810	3,538.0000	1,535.5000		0.0147	0.0233	6.1594	60.8252
	SD	0.2828	0.1414	0.7071	0.9899	0.0884	0.6364	0.3889	0.1202	0.0071	0.0495	0.0057	0.0714		0.8973	0.0424	261.6295	65.7609		0.0013	0.0013	0.6572	28.2547
**TDO2**	Mean	0.1800	0.1830	1.0920	3.1900	2.3250	1,118.5000	0.0331	0.7135	0.0935	0.0109	0.0142	0.0137		2.6215	2.8300	0.4400	102,250.0000	0.0104	0.0000	0.0031	0.0205	0.0429
	SD	0.0424	0.0198	0.1386	0.1556	0.4455	30.4056	0.0035	0.7587	0.0332	0.0081	0.0075	0.0075		3.2506	0.2828	0.0834	2,192.0310	0.0435	0.0000	0.0001	0.0012	0.0046
**WARS**	Mean	1,033.0000	53,985.0000	5,062.5000	1,001.5000	14,830.0000	90.4000	332.5000	5,552.0000		199.0000	2,355.0000	182.0000			495.5000	270.5000	59.4500		80.7000	100.0000		
	SD	42.4264	1,110.1576	267.9935	48.7904	56.5685		170.4127	60.8112		5.6569	165.4630	9.8995			3.5355	12.0208	4.8790		0.5657	0.0000		
**STAT1**	Mean	691.0000	7,426.0000	8,150.0000	896.5000	983.0000	88.8000	261.0000	822.0000		40.3500	959.0000	587.5000			214.0000	142.0000	2,192.0000		171.0000	543.0000		
	SD	16.9706	576.9991	200.8183	330.2189	22.6274		106.0660	15.5563		1.9092	7.0711	30.4056			1.4142	0.0000	101.8234		0.0000	21.2132		
**PIK3**	Mean	47.8500	43.9500	87.2000	1,805.0000	24.5500	42.8000	15.3500	6.6200		49.9000	40.0000	46.3500			724.0000	609.5000	266.5000		86.4500	94.1500		
	SD	1.3435	3.7477	1.8385	1,349.1597	0.0707		6.7175	0.5798		0.1414	2.1213	2.3335			1.4142	17.6777	6.3640		2.7577	4.8790		

TABLE S2 – Quantitative real–time RT-PCR analysis of total RNA from human and mouse MSCs, NSCs and tissues in the absence or presence of IFN-γ (100 IU/ml) or IFN-β (2,000 IU/ml) for 72 hours. The gene/β-actin ratios were multiplied by 10,000 for clarity purposes (except for the NSC results). Data are mean ± standard deviation (SD).
