# Supplementary material for: Interferon-γ Regulates the Proliferation and Differentiation of Mesenchymal Stem Cells via Activation of Indoleamine 2,3 Dioxygenase (IDO)
Source: PLoS One. 2011 Feb 16;6(2):e14698. doi: 10.1371/journal.pone.0014698 (PMC3040184; doi:10.1371/journal.pone.0014698)
Supplement: Table S3 — (0.07 MB PDF) [file pone.0014698.s008.pdf]

		Diff 5 days Control	Diff 5 days IFN-β 2000IU/ml	Diff 5 days IFN-γ 100IU/ml	Diff 5 days IFN-γ 100IU/ml +NH 15μM	Diff 5 days IFN-γ 100IU/ml +D1MT100μM	Diff 5 days IFN-γ 100IU/ml +L1MT100μM	Diff 5 days IFN-γ 100IU/ml +D1MT/L1MT100μM	Non Diff 3 days Control	Non Diff 3 days IFN-γ 100IU/ml	Non Diff 3 days IFN-γ 100IU/ml +D1MT/L1MT100μM
**CNP1**	Mean	255.667	388.667	543.667	523.000	468.333	486.000	665.000	51.033	133.333	100.200
	SEM	2.404	0.667	12.811	12.490	12.772	6.928	27.221	1.581	4.631	1.332
**GalC**	Mean	135.333	55.267	126.333	113.000	97.667	108.333	160.000	15.567	13.967	12.167
	SEM	1.202	0.120	2.906	2.646	2.669	1.453	6.429	0.498	0.491	0.133
**GFAP**	Mean	0.852	0.436	0.793	0.747	1.253	1.630	1.720	0.018	0.029	0.022
	SEM	0.008	0.001	0.019	0.017	0.035	0.023	0.070	0.001	0.001	0.000
**MAP2**	Mean	1.133	12.900	15.267	14.467	12.733	9.157	9.960	0.082	0.510	0.232
	SEM	0.012	0.000	0.353	0.353	0.348	0.130	0.416	0.003	0.018	0.003
**Nestin**	Mean	56.100	60.633	33.200	39.367	28.733	31.167	38.467	1.713	0.760	0.512
	SEM	0.513	0.133	0.757	0.921	0.784	0.433	1.581	0.052	0.026	0.007
**Id2**	Mean	809.333	239.667	229.667	186.333	198.000	212.000	232.667	82.267	185.667	163.333
	SEM	6.888	0.667	5.239	4.410	5.508	2.887	9.387	2.520	6.386	2.186
**NG2**	Mean	6.837	4.610	3.960	4.320	3.113	3.087	5.073	6.553	2.260	1.573
	SEM	0.060	0.010	0.093	0.104	0.084	0.043	0.211	0.202	0.078	0.022
**Hes1**	Mean	136.333	132.333	215.667	239.667	0.000	514.667	152.000	7.440	1.463	0.834
	SEM	1.202	0.333	5.239	5.608	0.000	7.219	6.429	0.229	0.052	0.011
**SCLIA1**	Mean	80.633	34.467	43.100	41.167	0.000	101.233	43.233	17.333	7.543	9.490
	SEM	0.689	0.067	0.987	0.977	0.000	1.534	1.784	0.524	0.261	0.121
**SCLIA3**	Mean	19.233	46.400	24.733	24.267	27.333	28.700	33.067	7.683	9.723	12.000
	SEM	0.176	0.100	0.555	0.561	0.726	0.404	1.374	0.238	0.347	0.153
**NPDC1**	Mean	53.133	19.033	40.533	34.900	28.600	25.833	62.000	10.417	11.333	12.100
	SEM	0.481	0.033	0.933	0.833	0.757	0.376	2.572	0.309	0.406	0.153
**GRM1**	Mean	1.900	0.799	1.720	1.603	1.400	3.747	3.057	0.419	0.439	0.591
	SEM	0.015	0.002	0.038	0.038	0.038	0.055	0.126	0.013	0.015	0.008
**Full IDO1**	Mean	2.520	99.900	59,193.330	52,456.670	47,130.000	60,070.000	69,833.330	0.021	11,763.330	10,753.330
	SEM	0.021	0.058	1,375.771	1,243.602	1,265.320	845.950	2,881.761	0.001	405.517	137.760
**Partial IDO1**	Mean	0.354	19.133	19,073.330	15,806.670	16,666.670	17,480.000	22,290.000	0.001	3,861.333	3,663.000
	SEM	0.003	0.033	442.016	375.604	447.002	245.425	918.768	0.000	133.849	46.822
**Full IDO2**	Mean	0.470	0.555	3.273	3.063	3.660	6.667	4.083	0.015	0.779	0.598
	SEM	0.004	0.001	0.078	0.074	0.099	0.095	0.167	0.000	0.027	0.008
**Partial IDO2**	Mean	0.141	0.405	2.113	2.137	1.893	3.720	1.277	0.000	0.644	0.522
	SEM	0.001	0.001	0.050	0.050	0.052	0.052	0.052	0.000	0.022	0.007

Table S3: Quantitative real–time RT-PCR analysis of neural marker expression by differentiated human MSCs. The gene/β-actin ratios were multiplied by 10,000 for clarity purposes. Data are mean ± standard error (SEM).
