# Supplementary material for: Interferon-γ Regulates the Proliferation and Differentiation of Mesenchymal Stem Cells via Activation of Indoleamine 2,3 Dioxygenase (IDO)
Source: PLoS One. 2011 Feb 16;6(2):e14698. doi: 10.1371/journal.pone.0014698 (PMC3040184; doi:10.1371/journal.pone.0014698)
Supplement: Table S4 — (0.08 MB PDF) [file pone.0014698.s009.pdf]

	IDO1		Oct--4		O4		O1		A2B5		MAP2		Nestin	
	Mean	SEM	Mean	SEM	Mean	SEM	Mean	SEM	Mean	SEM	Mean	SEM	Mean	SEM
**hMSC Control non differentiated**	51.0	12.6	66.3	16.6	16,330.6	10,120.6	145.4	33.4			43.8	20.4		
**hMSC 0/0**	320.8	198.4	153.6	94.4	903.7	427.8	598.6	179.3	3,905.5	1,810.8	80.4	11.8		
**hMSC IFN-γ/0**	8,085.8	2,354.5	265.5	153.3	885.9	304.2	4,448.5	2,360.6	10,251.6	2,689.6	693.4	93.0		
**hMSC IFN-γ/NH**	2,543.5	490.7	331.0	90.2	9,655.3	3,861.0	1,120.0	506.4	21,589.5	7,554.5	157.4	20.1		
**hMSC IFN-γ/DMT**	9,594.4	1,407.5	175.6	22.0	1,350.6	367.1	2,869.0	1,696.6	13,984.9	4,117.5	390.0	91.7		
**hMSC IFN-γ/LMT**	17,782.8	7,611.9	909.5	125.6	1,962.2	707.8	1,989.9	672.3	35,566.5	9,198.6	608.1	248.7		
**mMSC 0/0**	175.1	49.7	5,153.7	5,066.7	37,206.6	13,897.4	17,724.1	9,786.5			331.4	81.8	2,480.6	787.1
**mMSC IFN-γ/0**	1,593.1	708.3	6,270.8	4,561.0	109,469.9	33,122.1	19,272.3	4,080.7			11,631.2	2,291.4	3,861.3	644.0
**mMSC IFN-γ/NH**	1,224.0	310.1	15,945.6	7,233.1	20,403.9	3,034.9	13,611.0	2,722.4			9,422.5	2,935.2	19,388.4	12,639.2
**mMSC IFN-γ/DMT**	1,064.7	179.6	6,452.9	4,375.9	9,006.5	1,047.9	25,269.9	8,014.5			3,290.9	595.0	9,643.6	6,333.4
**mMSC IFN-γ /LMT**	2,388.4	1,462.9	17,601.6	7,904.5	80,432.3	17,880.0	45,402.6	3,775.5			11,741.2	2,983.3	7,371.5	1,001.7

Table S4: Quantitative analysis of IDO1 and neural marker expression by differentiated mouse and human MSCs as revealed by immunostaining. Cells were cultured in the neural differentiation media together with 100 IU/ml IFN-γ and/or IDO inhibitors norharmane (15 μM), D-1-methyl-tryptophan (100 μM) and L-1-methyl-tryptophan (100 μM). Data are mean ± standard error (SEM). Images were taken under identical exposure conditions. The density of immunostaining normalised to the number of nuclei are represented as means ± SEM of at least three independent experiments. Abbreviations: IDO1, indoleamine 2,3-dioxygenase 1; IFN-γ, interferon-γ; MAP2, microtubule-associated protein 2; D-1MT, D-1-methyl-tryptophan; L-1MT, L-1-methyl-tryptophan.
